# Facet sensitivity of iron carbides in Fischer-Tropsch synthesis

**DOI:** 10.1038/s41467-024-50544-1

**Published:** 2024-07-19

**Authors:** Wenlong Wu, Jiahua Luo, Jiankang Zhao, Menglin Wang, Lei Luo, Sunpei Hu, Bingxuan He, Chao Ma, Hongliang Li, Jie Zeng

**Affiliations:** 1 School of Chemistry & Chemical Engineering, Anhui University of Technology, Ma’anshan, Anhui 243002 P. R. China; 2 Hefei National Research Center for Physical Sciences at the Microscale, Key Laboratory of Strongly-Coupled Quantum Matter Physics of Chinese Academy of Sciences, Key Laboratory of Surface and Interface Chemistry and Energy Catalysis of Anhui Higher Education Institutes, Department of Chemical Physics, University of Science and Technology of China, Hefei, Anhui 230026 P. R. China; 3 National Synchrotron Radiation Laboratory, University of Science and Technology of China, Hefei, Anhui 230026 P. R. China; 4 College of Materials Science and Engineering, Hunan University, Changsha, 410082 P. R. China

**Keywords:** Heterogeneous catalysis, Nanoparticles, Catalyst synthesis

## Abstract

Fischer-Tropsch synthesis (FTS) is a structure-sensitive reaction of which performance is strongly related to the active phase, particle size, and exposed facets. Compared with the full-pledged investigation on the active phase and particle size, the facet effect has been limited to theoretical studies or single-crystal surfaces, lacking experimental reports of practical catalysts, especially for Fe-based catalysts. Herein, we demonstrate the facet sensitivity of iron carbides in FTS. As the prerequisite, {202} and {112} facets of χ-Fe_5_C_2_ are fabricated as the outer shell through the conformal reconstruction of Fe_3_O_4_ nanocubes and octahedra, as the inner cores, respectively. During FTS, the activity and stability are highly sensitive to the exposed facet of iron carbides, whereas the facet sensitivity is not prominent for the chain growth. According to mechanistic studies, {202} χ-Fe_5_C_2_ surfaces follow hydrogen-assisted CO dissociation which lowers the activation energy compared with the direct CO dissociation over {112} surfaces, affording the high FTS activity.

## Abstract (web summary)

The impact of facets on Fischer-Tropsch synthesis has primarily been explored through theoretical studies or on single-crystal surfaces, lacking experimental data on practical catalysts. Here, the authors provide experimental evidence of the facet sensitivity of iron carbides during syngas conversion by creating {202} and {112} χ-Fe_5_C_2_ facets.

## Introduction

Fischer-Tropsch synthesis (FTS) is a structure-sensitive reaction for the sustainable production of synthetic fuels and building-block chemicals from syngas^1–6^. The catalytic performance is strongly related to the active phase, particle size, and exposed facets of the active components such as iron, cobalt, and ruthenium^7–13^. Compared with Co and Ru, Fe-based catalysts have superior properties including resistance to the formation of methane, low cost, high adaptability to broad H_2_/CO ratios, and versatility to various useful products^14–16^. For Fe-based catalysts, pure-phase iron carbides were synthesized by using Fe(CO)_5_ reagent, which was explored by means of in-situ characterizations^17,18^. The Fe_3_O_4_@χ-Fe_5_C_2_ core-shell catalysts were constructed by pyrolyzing iron-containing metal-organic frameworks, whereas the obtained nanoparticles were irregular spherical particles^19^. The transformation of reduced iron phases to iron carbides promoted the formation of hydrocarbon species in FTS^20^. The effects of the active phase and particle size have been extensively studied^19,21–26^. These effects are generally entangled with the contributions of surface terrace, corner, edge, and step-edge sites, where differences in coordination numbers and surface topology may lead to substantial differences in intrinsic performance^27^. Up to date, the investigation of the facet effect has been limited to theoretical calculations or single-crystal surfaces. For instance, density functional theory (DFT) calculations of CO activation on χ-Fe_5_C_2_ surfaces indicated that the terraced (510) surface inclined to directly dissociate CO molecules, whereas the stepped (010) and (001) surfaces preferred the hydrogen-assisted CO dissociation route^28^. In-situ scanning tunneling microscopy visualized on-surface ethylene polymerization on a carburized Fe(110) single-crystal surface^29^. However, there is no experimental report on the facet effect of practical Fe-based catalysts due to the complexity and dynamic structural evolution of iron carbides during FTS.

As the widely accepted active phase for FTS, *χ*-Fe_5_C_2_ has a base-centered monoclinic (*bcm*) structure with space group C2/*c* (*a* = 11.56 Å, *b* = 4.57 Å, *c* = 5.06 Å, and *β* = 97.74°)^30,31^. Owing to the low symmetry of the lattice structure, it remains as a grand challenge to synthesize *χ*-Fe_5_C_2_ nanocrystals with uniformly exposed surfaces. We proposed to use a highly symmetrical template as the core to support the *χ*-Fe_5_C_2_. Fe_3_O_4_ has a face-centered cubic (*fcc*) structure with a high symmetry. Herein, we reported the conformal reconstruction of well-defined Fe_3_O_4_ nanocrystals to generate χ-Fe_5_C_2_ with specifically exposed surfaces. The samples consisted of an inner core of Fe_3_O_4_ and an outer shell of χ-Fe_5_C_2_, denoted as Fe_3_O_4_@χ-Fe_5_C_2_ nanocrystals. We obtained Fe_3_O_4_@χ-Fe_5_C_2_ nanocrystals with surfaces terminated in {202} and {112} facets of χ-Fe_5_C_2_ shells through using cubic and octahedral Fe_3_O_4_ as the templates, respectively (Fe_3_O_4_@χ-Fe_5_C_2_ nanocubes and octahedra, respectively). We discovered that Fe_3_O_4_@χ-Fe_5_C_2_ nanocubes were more catalytically active and stable than the octahedral counterpart during FTS, whereas the facet sensitivity was not prominent for the chain growth. According to mechanistic studies, the high activity of {202} χ-Fe_5_C_2_ surfaces derived from the unique reaction path in which the hydrogen-assisted CO dissociation route lowered the activation energy relative to the direct CO dissociation route over {112} surfaces.

## Results and discussion

### Synthesis and characterization of Fe_3_O_4_@χ-Fe_5_C_2_ nanocubes

**Fig. 1:**
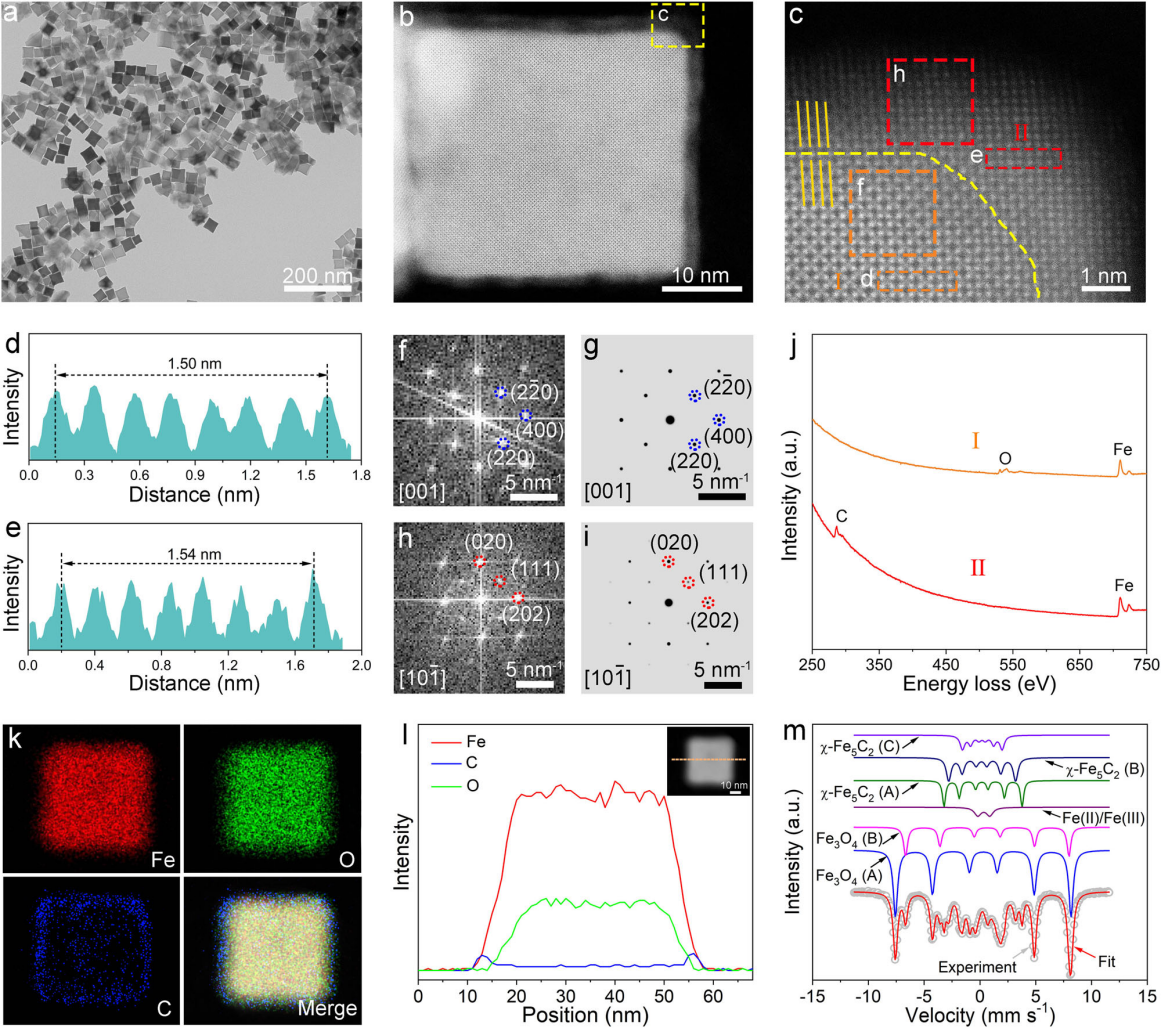
Structural characterizations of Fe_3_O_4_@χ-Fe_5_C_2_ nanocubes. **a** TEM image of Fe_3_O_4_@χ-Fe_5_C_2_ nanocubes. **b** HAADF-STEM image of an individual Fe_3_O_4_@χ-Fe_5_C_2_ nanocube. **c** Magnified HAADF-STEM image of the region marked by the corresponding box in panel (**b**). **d** Intensity profile recorded from the area indicated by the rectangular box in panel (**c**). **e** Intensity profile recorded from the area indicated by the rectangular box in panel (**c**). **f** FFT pattern from box **f** in panel (**c**). **g** Simulated FFT pattern of Fe_3_O_4_ along the [001] direction. **h** FFT pattern from box **h** in panel (**c**). **i** Simulated FFT pattern of χ-Fe_5_C_2_ along the [10-1] direction. **j** EELS spectra of a Fe_3_O_4_@χ-Fe_5_C_2_ nanocube in panel (**c**). **k** Elemental mapping images of a Fe_3_O_4_@χ-Fe_5_C_2_ nanocube. **l** Line-scan profile recorded along the line of the inset HAADF image. **m** Mössbauer spectra of Fe_3_O_4_@χ-Fe_5_C_2_ nanocubes.

To begin with, Fe_3_O_4_ nanocubes were prepared with an average size of 40.5 ± 3.9 nm and a purity of 95.3% (Supplementary Fig. 1). Fe_3_O_4_@χ-Fe_5_C_2_ core-shell nanocubes were synthesized via surface reconstruction of Fe_3_O_4_ under syngas atmosphere. Specifically, Fe_3_O_4_ nanocubes were reduced under 1 bar of H_2_ with a gas-flow rate of 100 mL min^−1^ at 270 °C for 10 h. This treatment ensured the removal of surface organic species (Supplementary Fig. 2). Afterwards, the obtained samples underwent surface reconstruction in a fixed-bed reactor under 20 bar of syngas (32 vol% H_2_, 64 vol% CO, and 4 vol% Ar) with a space velocity of 2400 mL h^−1^ g_cat_^−1^ at 270 °C for 20 h. Figure 1a shows the transmission electron microscopy (TEM) image of Fe_3_O_4_@χ-Fe_5_C_2_ nanocubes. The average size of Fe_3_O_4_@χ-Fe_5_C_2_ nanocubes was 40.1 ± 3.8 nm with a purity of 90.0% (Supplementary Fig. 3). The Fe_3_O_4_@χ-Fe_5_C_2_ nanocubes exhibited a surface area of 20.4 m^2^ g^−1^ based on the Brunauer-Emmett-Teller (BET) method (Supplementary Fig. 4a). The high-angle annular dark-field scanning transmission electron microscopy (HAADF-STEM) image taken from one of the nanocubes revealed a periodic lattice extending across the entire surface (Fig. 1b). The magnified image clearly revealed lattice differences between the edge region and the central region, implying the formation of the core-shell structure (Fig. 1c). In the case of nanocrystals with a core-shell structure, a slight lattice mismatch (*f* < ~5%) is required for the epitaxial surface layer to form over the inner core^32^. This epitaxial relationship allows for the maintenance of orientation between the growth layer and the substrate within the first few atomic layers. The spacing of Fe_3_O_4_(400) planes (0.21 nm) in the core was approximately equal to that of χ-Fe_5_C_2_(202) planes (0.22 nm) in the shell (Fig. 1d, e). The small lattice mismatch (*f*) of 4.65% calculated from Eq. (1) ensures the preservation of the epitaxial orientation relationship.

1 $$f=2\times {{{\rm{|}}}}{d}_{{{\rm{shell}}}}-{d}_{{{\rm{core}}}}{{{\rm{|}}}}/({d}_{{{\rm{shell}}}}+{d}_{{{\rm{core}}}})$$

In Eq. (1), *d*_shell_ and *d*_core_ refer to the lattice spacings of the shell and the core, respectively. The small lattice mismatch results in only one type of facet at the surface layer of Fe_3_O_4_@χ-Fe_5_C_2_ nanocubes. To identify the corresponding facets, we conducted the fast Fourier transform (FFT) analysis. According to the combination of the experimental and simulated FFT patterns, the region in the inner core was indexed as the (400) facet of *fcc* Fe_3_O_4_ along the [001] zone axis, while that in the outer shell corresponded to the (202) facet of *bcm* χ-Fe_5_C_2_ along the [10−1] zone axis (Fig. 1f–i). The HAADF-STEM images of different corners and edges in an individual nanoparticle indicated that the surfaces exposed a uniform χ-Fe_5_C_2_ shell with the {202} facets (Supplementary Fig. 5). The average thickness of the shell was determined as 2.0 nm, approximating ten atomic layers (Supplementary Fig. 5).

The core of iron oxides and the shell of iron carbides were further confirmed through the spatial elemental analysis. Specifically, the electron energy loss spectroscopy (EELS) image implied that the core region mainly comprised Fe and O elements while the shell region contained Fe and C elements (Fig. 1j). The scanning transmission electron microscopy-energy dispersive X-ray (STEM-EDX) analysis including the elemental mapping images and the line-scanning profile confirmed that O and C elements were mainly located in the core and the shell, respectively, while Fe was homogeneously distributed throughout the particle (Fig. 1k, l).

To quantify the contents of different phases in Fe_3_O_4_@χ-Fe_5_C_2_ nanocubes, we carried out Mössbauer and X-ray diffraction (XRD) characterizations. Based on the Mössbauer results, the sample was composed of 63.4 wt% of Fe_3_O_4_, 33.2 wt% of χ-Fe_5_C_2_, and 3.4 wt% of Fe(II)/Fe(III) species (Fig. 1m and Supplementary Table 1). Fe(II)/Fe(III) species indicated the presence of some poorly crystallized iron oxides. The quantitative analysis of XRD showed that χ-Fe_5_C_2_ occupied 29.8 wt% of the total mass, approaching that (33.2 wt%) obtained from the Mössbauer spectra (Supplementary Fig. 6a and Supplementary Table 2).

### Evolution from Fe_3_O_4_ nanocubes to Fe_3_O_4_@χ-Fe_5_C_2_ nanocubes

**Fig. 2:**
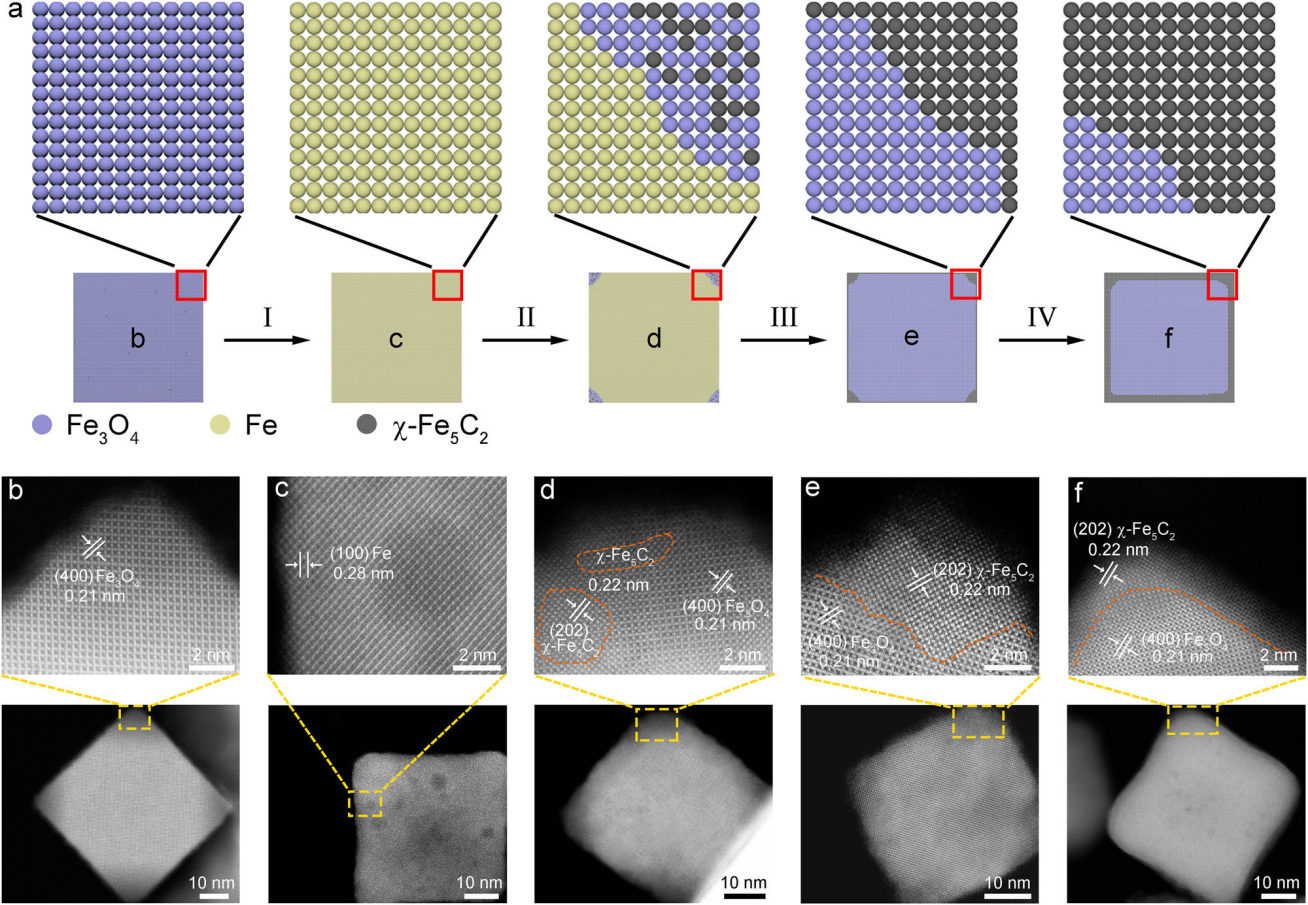
Structural characterizations of the evolution from the Fe_3_O_4_ nanocube to the Fe_3_O_4_@χ-Fe_5_C_2_ nanocube. **a** Schematic of the major steps involved in the continuous evolution from the Fe_3_O_4_ nanocube to the Fe_3_O_4_@χ-Fe_5_C_2_ nanocube. I refers to the reduction process; II refers to the corner carburization and oxidation; III refers to the surface carburization, O migration, and bulk oxidation; IV refers to the surface balanced carburization and oxidation. **b** HAADF-STEM images of an individual Fe_3_O_4_ nanocube. **c** HAADF-STEM images of an individual Fe nanocube. **d**–**f** HAADF-STEM images of an individual nanocrystal after the treatment of Fe nanocubes with syngas for 2, 5, and 10 h, respectively.

Figure 2a depicts the schematic of the evolution from Fe_3_O_4_ nanocubes to Fe_3_O_4_@χ-Fe_5_C_2_ nanocubes. The major steps were verified by HAADF-STEM images and XRD patterns. Specifically, after reduction, the initial Fe_3_O_4_ nanocubes with {400} facets were transformed into metallic Fe nanocubes with their surfaces terminated in {100} facets (Fig. 2b, c and Supplementary Fig. 6b, c). When metallic Fe nanocubes were exposed to the syngas, the thermodynamic driving force induced the phase transition from metallic Fe to iron carbides. The surface Fe atoms underwent carburization and oxidation by reacting with carbon and oxygen species derived from the dissociated CO. After the exposure to syngas for 2 h, the corners were preferentially carburized and oxidized, resulting in the random distribution of Fe_3_O_4_ and χ-Fe_5_C_2_ domains (Fig. 2d). Notably, the diffractions of Fe_3_O_4_ and metallic Fe were clearly observed, whereas the crystalline χ-Fe_5_C_2_ was not detected by XRD (Supplementary Fig. 6d). This result implied that χ-Fe_5_C_2_ existed in short-range order with an amorphous structure. When Fe nanocubes were exposed to syngas for 5 h, carbon atoms cleaved from CO completely carburized the metallic Fe at the corner of the nanocube, while the dissociated oxygen atoms permeated into the interior, resulting in the bulk transition into Fe_3_O_4_ (Fig. 2e). At this stage, the diffractions of χ-Fe_5_C_2_ were observed in the XRD patterns, along with metallic Fe and Fe_3_O_4_ (Supplementary Fig. 6e). When Fe nanocubes were exposed to syngas for 10 h, the dissociated carbon atoms carburized the surface layers from the corners to the whole faces (Fig. 2f and Supplementary Fig. 6f). A core-shell structure with the Fe_3_O_4_ core and χ-Fe_5_C_2_ surface formed as the result of that the nanocubes lost the thermodynamic driving force for further carburization or oxidation^33^. The core-shell structure did not show obvious change when the syngas treatment was prolonged to 20 h (Fig. 1b, c). When the Fe_3_O_4_@χ-Fe_5_C_2_ core-shell structure formed at a steady state, the excess dissociated oxygen atoms were released into the gas phase in the form of CO_2_ and H_2_O to prevent the oxidation of the shell. The excess dissociated carbon atoms reacted with surface-dissociated hydrogen atoms to yield hydrocarbons rather than to permeate and carburize the Fe_3_O_4_ interlayer. The stable core-shell structure was the result of the dynamic balance of the hydrocarbon production, surface oxidation, and carburization in the syngas environment.

We conclude the driving force to limit a single type of phase and facet at the surface layer as follows. The driving force to regulate the phase is the carbon chemical potential of reaction conditions. It was reported that the activation energy for carbon diffusion in Fe (43.9–69.0 kJ mol^−1^) was lower than that for the FTS reaction (89.1 ± 3.8 kJ mol^−1^)^14^. Consequently, surface carbon atoms cleaved from CO exhibit a pronounced affinity to Fe atoms, thereby instigating a phase transition from iron to FeC_x_ during the initial stage of the FTS reaction. Upon the formation of active FeC_x_ on the surface, the FTS reaction is facilitated, resulting in the release of oxygen species, predominantly in the form of H_2_O, into the gas phase. This influx of H_2_O induces Fe oxide formation and impedes further carburization. As the concentration of oxygen species diminishes in the gas phase, a greater quantity of carbide accumulates on the surface, pushing the reaction condition back to the equilibrium position and vice versa. As the inner core of the catalyst oxidizes to Fe_3_O_4_, it becomes more resistant to carbon permeation and carburization compared to metallic Fe. According to carbon chemical potential theory, carbon-rich χ-Fe_5_C_2_ is the thermodynamically stable phase under our reaction condition (20 bar, CO:H_2_ = 1:2, 270 °C)^21^. As such, other Fe carbides present in the surface layers will evolve into χ-Fe_5_C_2_ as the reaction progresses. Moreover, the uniform facet of the substrate and the small lattice mismatch (<~5%) within the core-shell structure ensure the preservation of the epitaxial orientation relationship.

### Synthesis and characterization of Fe_3_O_4_@χ-Fe_5_C_2_ octahedra

**Fig. 3:**
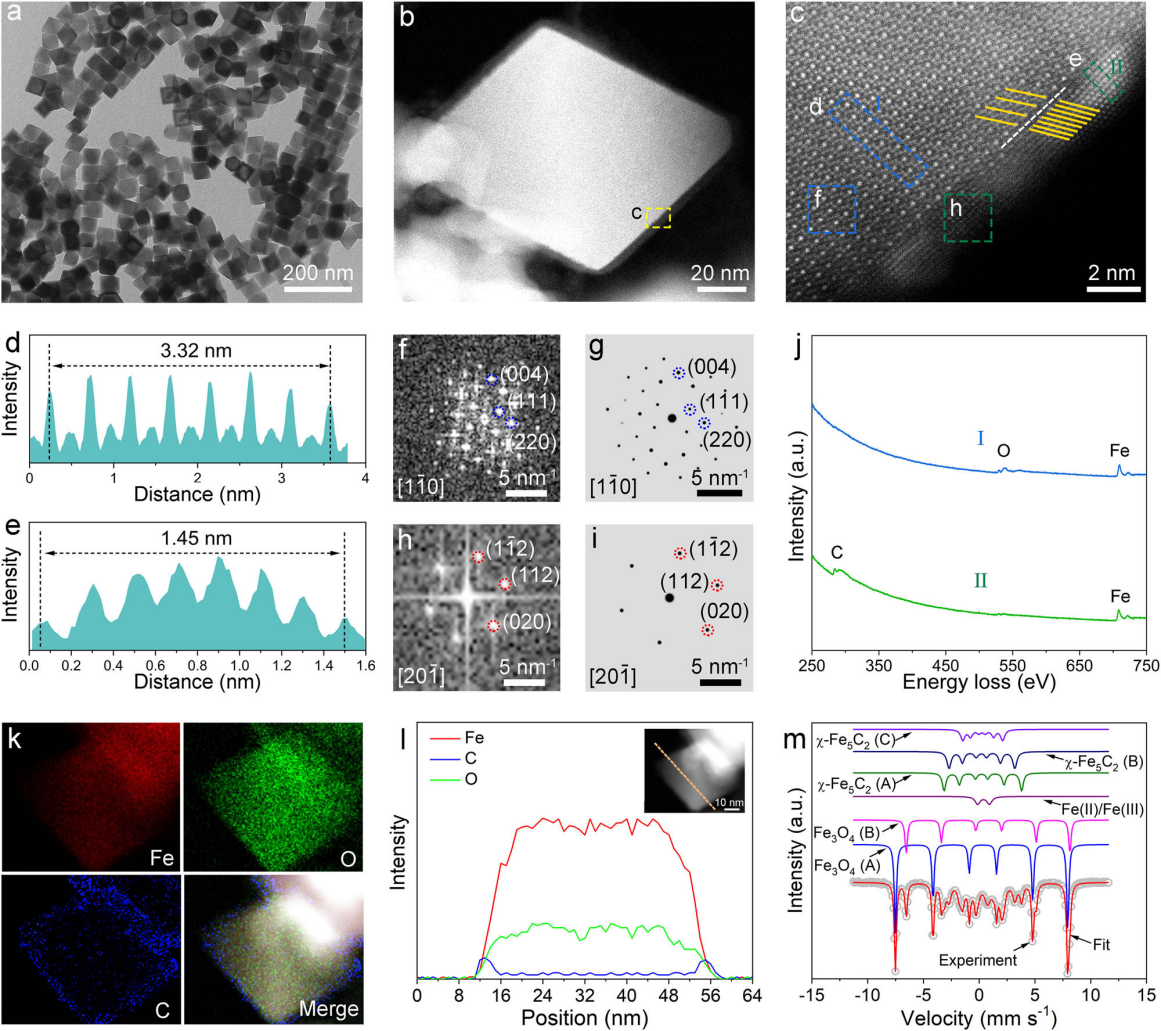
Structural characterizations of Fe_3_O_4_@χ-Fe_5_C_2_ octahedra. **a** TEM image of Fe_3_O_4_@χ-Fe_5_C_2_ octahedra. **b** HAADF-STEM image of an individual Fe_3_O_4_@χ-Fe_5_C_2_ octahedron. **c** Magnified HAADF-STEM image of the region marked by the corresponding box in panel (**b**). **d** Intensity profile recorded from the area indicated by the rectangular box in panel (**c**). **e** Intensity profile recorded from the area indicated by the rectangular box in panel (**c**). **f** FFT pattern from box **f** in panel (**c**). **g** Simulated FFT pattern from of Fe_3_O_4_ along the [1−10] direction. **h** FFT pattern from box **h** in panel (**c**). **g** Simulated FFT pattern from of χ-Fe_5_C_2_ along the [20−1] direction. **j** EELS spectra of a Fe_3_O_4_@χ-Fe_5_C_2_ octahedron in panel (**c**). **k** elemental mapping images of a Fe_3_O_4_@χ-Fe_5_C_2_ octahedron. **l** Line-scan profile recorded along the line of the inset HAADF image. **m** Mössbauer spectra of Fe_3_O_4_@χ-Fe_5_C_2_ octahedra.

For comparison, we applied the surface reconstruction procedure to prepare Fe_3_O_4_@χ-Fe_5_C_2_ octahedra. We carbonized the synthesized Fe_3_O_4_ octahedra following a similar approach to that of nanocubes (Supplementary Fig. 7). The average size of Fe_3_O_4_@χ-Fe_5_C_2_ octahedra was 45.4 ± 3.5 nm with a purity of 93.3% (Fig. 3a and Supplementary Fig. 8). The surface area of Fe_3_O_4_@χ-Fe_5_C_2_ octahedra was 22.0 m^2^ g^−1^ based on the BET method (Supplementary Fig. 4b). The lattice disparity between the core and the shell was clearly revealed by the HAADF-STEM images (Fig. 3b and Supplementary Fig. 9). The uniform shell had an average thickness of 1.7 nm, corresponding to eight atomic layers (Fig. 3c). We assigned the lattice parameters with the help of the experimental and simulated FFT patterns. Specifically, the inner core took a lattice parameter of 0.48 nm which was indexed as the (111) facet of *fcc* Fe_3_O_4_ along the [001] zone axis (Fig. 3d, f, g). For the outer shell, the lattice parameter of 0.21 nm was assigned to the (112) facet of *bcm* χ-Fe_5_C_2_ along the [10-1] zone axis (Fig. 3e, h, i). Besides lattice matching, the epitaxial orientation can also be preserved via domain matching, where the spacing of *m* lattice planes in the epilayer is approximately equal to *n* in the substrate^32,34,35^. We observed that the spacing of three Fe_3_O_4_(111) planes in the core was approximately equal to that of seven χ-Fe_5_C_2_(112) planes in the shell (Fig. 3c). Such periodicity leads to a commensurate epitaxial relationship with a low mismatch value of 2.06% according to Eq. (2).

2 $$f=2\times {{{\rm{|}}}}7\times {d}_{{{\rm{shell}}}}-3\times {d}_{{{\rm{core}}}}{{{\rm{|}}}}/(7\times {d}_{{{\rm{shell}}}}+3\times {d}_{{{\rm{core}}}})$$

The domain match allows the conformal growth for the (112) facet of χ-Fe_5_C_2_ over the (111) facet of Fe_3_O_4_. The distribution of Fe_3_O_4_ at the core and χ-Fe_5_C_2_ at the shell was supported by the EELS image, STEM-EDX elemental mapping images, and line scanning profile (Fig. 3j–l). Mössbauer result indicated that Fe_3_O_4_@χ-Fe_5_C_2_ octahedra contained 65.1 wt% of Fe_3_O_4_ and 29.5 wt% of χ-Fe_5_C_2_ (Fig. 3m). The content of χ-Fe_5_C_2_ was consistent with the XRD quantitative analysis result (27.6%) (Supplementary Tables 1 and 2).

### Facet effect on activity and selectivity

**Fig. 4:**
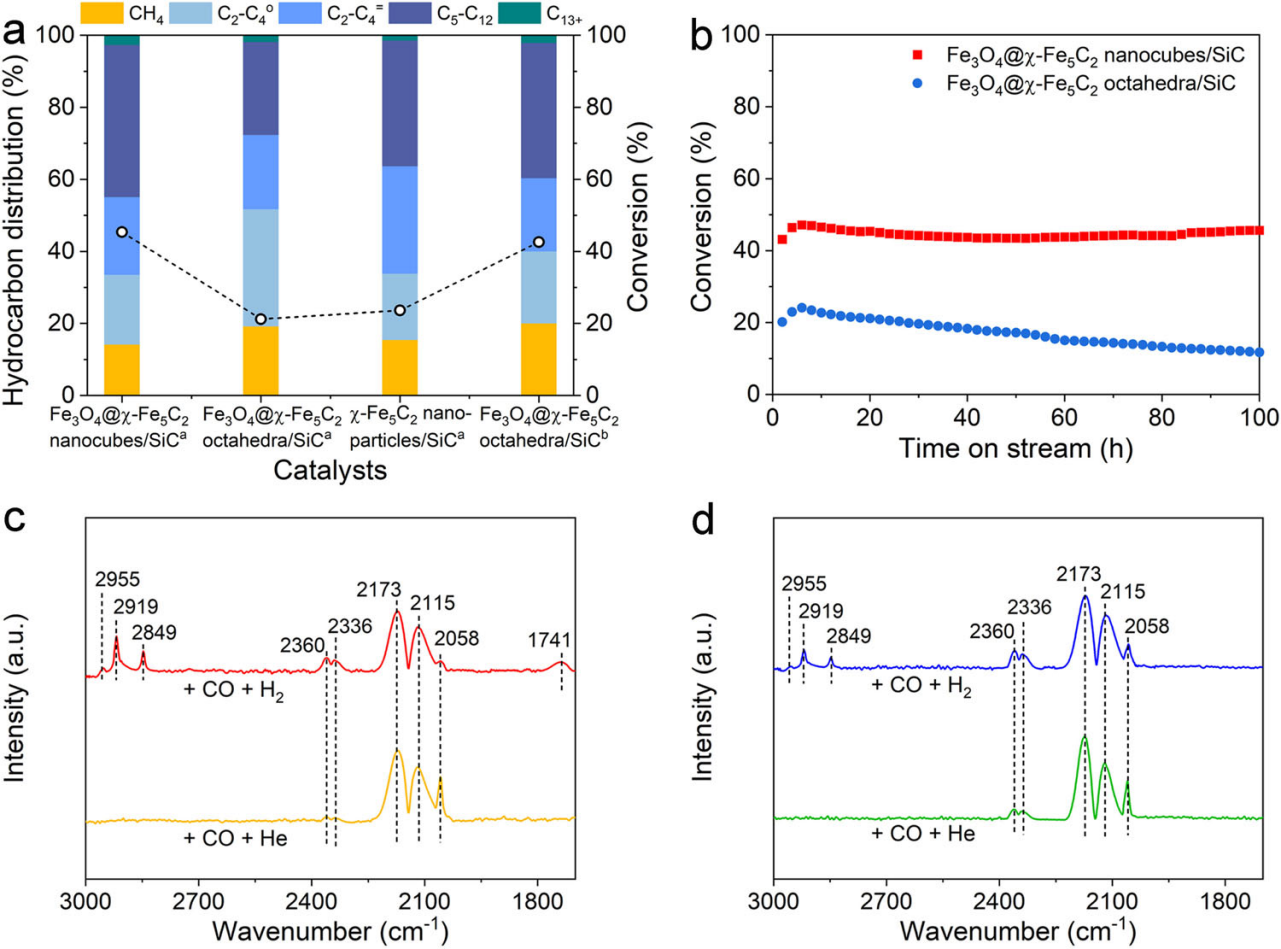
Catalytic properties and structural characterizations of Fe_3_O_4_@χ-Fe_5_C_2_ nanocubes/SiC and octahedra/SiC. **a** CO conversion and selectivity of Fe_3_O_4_@χ-Fe_5_C_2_ nanocubes/SiC, Fe_3_O_4_@χ-Fe_5_C_2_ octahedra/SiC, and χ-Fe_5_C_2_/SiC. ^a^ refers to that the reaction was conducted under 20 bar of syngas (CO:H_2_ = 1:2, 2400 mL h^−1^ g_cat_^−1^) at 270 °C. ^b^ refers to that the reaction was conducted under 20 bar of syngas (CO:H_2_ = 1:2, 800 mL h^−1^ g_cat_^−1^) at 270 °C. **b** Stability tests of Fe_3_O_4_@χ-Fe_5_C_2_ nanocubes/SiC and octahedra/SiC. The reaction was conducted under 20 bar of syngas (CO:H_2_ = 1:2, 2400 mL h^−1^ g_cat_^−1^) at 270 °C. **c** In-situ DRIFTS spectra of Fe_3_O_4_@χ-Fe_5_C_2_ nanocubes and **d** Fe_3_O_4_@χ-Fe_5_C_2_ octahedra after being exposed to CO for 30 min and purged with He or H_2_ for 30 min at 270 °C.

We explored the facet effect of iron carbides on FTS properties. As the shells of Fe_3_O_4_@χ-Fe_5_C_2_ nanocrystals are more than six atomic layers thick, the electronic coupling between the core and outermost layer in the shell is essentially lost, and thus the ability to access the strain-dependent catalytic activity will be gone^36^. Besides, it was worth noting that no promoters or additives were added since the purpose of this work was to investigate the intrinsic catalytic performance of different exposed facets of χ-Fe_5_C_2_. Fe_3_O_4_@χ-Fe_5_C_2_ nanocubes and octahedra were loaded on the SiC support, denoted as Fe_3_O_4_@χ-Fe_5_C_2_ nanocubes/SiC and octahedra/SiC, respectively. The TEM images of these nanocrystals and the corresponding particle models from different orientations were shown in Supplementary Figs. 10 and 11. The catalytic properties of Fe_3_O_4_@χ-Fe_5_C_2_ nanocubes/SiC and octahedra/SiC were evaluated in a fixed-bed reactor under 20 bar of syngas (64 vol% H_2_, 32 vol% CO, and 4 vol% Ar) with a space velocity of 2400 mL h^−1^ g_cat_^−1^ at 270 °C, denoted as the standard condition. The CO conversion of Fe_3_O_4_@χ-Fe_5_C_2_ nanocubes/SiC was 45.4%, which was higher than that (21.2%) of Fe_3_O_4_@χ-Fe_5_C_2_ octahedra/SiC (Fig. 4a and Supplementary Table 3).

To compare the catalytic activity more accurately, we calculated the TOF numbers based on the moles of CO converted per mole of surface Fe atoms per hour. The moles of Fe atoms on the surface of Fe_3_O_4_@χ-Fe_5_C_2_ nanocrystals were determined by CO pulse chemisorption measurement. The moles of Fe atoms on the surface of Fe_3_O_4_@χ-Fe_5_C_2_ nanocubes/SiC was 24.1 µmol g^−1^, higher than that (19.5 µmol g^−1^) of Fe_3_O_4_@χ-Fe_5_C_2_ octahedra/SiC (Supplementary Fig. 12a, b). The TOF number of Fe_3_O_4_@χ-Fe_5_C_2_ nanocubes/SiC was 645.9 h^−1^, being 1.7 times as high as that (372.7 h^−1^) of Fe_3_O_4_@χ-Fe_5_C_2_ octahedra/SiC. The carbon balance value of Fe_3_O_4_@χ-Fe_5_C_2_ nanocubes/SiC was 98.7%, similar to that (96.5%) of Fe_3_O_4_@χ-Fe_5_C_2_ octahedra/SiC (Supplementary Table 3). The high carbon balance value indicated that the different CO conversions were not caused by continuous carburization. For reference, we prepared pure χ-Fe_5_C_2_ nanoparticles which mainly exposed the thermodynamically most stable {510} facet through the wet-chemical route^30^ (Supplementary Fig. 13). The CO conversion of χ-Fe_5_C_2_ nanoparticles/SiC was 23.6% (Fig. 4a and Supplementary Table 3). As such, the {202} χ-Fe_5_C_2_ facet exposed on Fe_3_O_4_@χ-Fe_5_C_2_ nanocubes/SiC was more active than the {112} χ-Fe_5_C_2_ facet exposed on Fe_3_O_4_@χ-Fe_5_C_2_ octahedra/SiC and thermodynamically stable surfaces of iron carbides.

With respect to the selectivity, the C_5+_ selectivity of Fe_3_O_4_@χ-Fe_5_C_2_ nanocubes/SiC was 44.8 C%, higher than that (27.6 C%) of Fe_3_O_4_@χ-Fe_5_C_2_ octahedra/SiC (Fig. 4a and Supplementary Table 3). The C_2_-C_4_ selectivity of Fe_3_O_4_@χ-Fe_5_C_2_ nanocubes/SiC was 41.0 C%, lower than that (53.1 C%) of Fe_3_O_4_@χ-Fe_5_C_2_ octahedra/SiC (Fig. 4a and Supplementary Table 3). Additionally, the ratio of olefins to paraffins (o/p ratio) among C_2_-C_4_ for Fe_3_O_4_@χ-Fe_5_C_2_ nanocubes/SiC was 1.1, higher than that (0.6) for Fe_3_O_4_@χ-Fe_5_C_2_ octahedra/SiC (Fig. 4a and Supplementary Table 3). The selectivity for C_2_-C_4_^=^ olefins over Fe_3_O_4_@χ-Fe_5_C_2_ nanocubes/SiC was 21.6 C%, approaching that (20.6 C%) over Fe_3_O_4_@χ-Fe_5_C_2_ octahedra/SiC (Supplementary Table 3). Additionally, the selectivity for C_5_–C_12_^=^ olefins over Fe_3_O_4_@χ-Fe_5_C_2_ nanocubes/SiC was 17.9 C%, higher than that (11.3 C%) over Fe_3_O_4_@χ-Fe_5_C_2_ octahedra/SiC (Supplementary Table 3). As for χ-Fe_5_C_2_ nanoparticles/SiC, the selectivities for CH_4_, C_2_-C_4_, C_5_-C_12_, and C_13+_ were 15.5 C%, 48.2 C%, 34.9 C%, and 1.4 C%, respectively (Fig. 4a and Supplementary Table 3). The C_2_-C_4_^=^ and C_5_-C_12_^=^ selectivities were 29.8 C% and 16.9 C%, respectively (Supplementary Table 3). Actually, the distribution of hydrocarbon products for Fe_3_O_4_@χ-Fe_5_C_2_ nanocubes/SiC, Fe_3_O_4_@χ-Fe_5_C_2_ octahedra/SiC, and χ-Fe_5_C_2_ nanoparticles/SiC followed a typical Anderson-Schulz-Flory (ASF) statistics. The probabilities of chain growth (*α*) for Fe_3_O_4_@χ-Fe_5_C_2_ nanocubes/SiC, Fe_3_O_4_@χ-Fe_5_C_2_ octahedra/SiC, and χ-Fe_5_C_2_ nanoparticles/SiC were calculated as 0.66, 0.62, and 0.63, respectively (Supplementary Figs. 14, 15). To compare the catalytic selectivity more appropriately, we adjusted the space velocity of Fe_3_O_4_@χ-Fe_5_C_2_ octahedra/SiC to keep the reaction over cubic and octahedral nanocrystals at similar conversion levels. When the space velocity was lowered to 800 mL h^−1^ g_cat_^−1^, CO conversion of Fe_3_O_4_@χ-Fe_5_C_2_ octahedra/SiC reached 42.6%, approaching that (45.4%) of the cubic counterpart under the standard condition (Fig. 4a and Supplementary Table 3). The C_5+_ selectivity of Fe_3_O_4_@χ-Fe_5_C_2_ octahedra/SiC was 39.6 C%, while the C_2_-C_4_ hydrocarbons occupied 40.3 C% of all hydrocarbons (Fig. 4a and Supplementary Table 3). The selectivities for C_2_-C_4_^=^ and C_5_-C_12_^=^ were 18.3 C% and 16.0 C%, respectively (Supplementary Table 3) The *α* value was calculated as 0.64 at this conversion level (Supplementary Fig. 16). Therefore, the effect of crystal face on selectivity is not as great as that on activity.

We evaluated the long-term stability of Fe_3_O_4_@χ-Fe_5_C_2_ nanocrystals/SiC through a 100-h test. The CO conversion of Fe_3_O_4_@χ-Fe_5_C_2_ nanocubes/SiC fluctuated within 1% during the whole test, indicating the high stability of this catalyst (Fig. 4b). The cubic morphology was preserved after 100 h on stream (Supplementary Fig. 17a, b). We measured the thickness of the shell layer for Fe_3_O_4_@χ-Fe_5_C_2_ nanocubes after the reaction. The thickness of the shell increased from 2.0 nm to 2.6 nm after the reaction (Fig. 1c and Supplementary Fig. 17c). The lattice parameter of the Fe_3_O_4_ inner core was measured as 0.21 nm, which was indexed as the (400) facet of Fe_3_O_4_ (Supplementary Fig. 17d). The lattice parameter of the χ-Fe_5_C shell was 0.22 nm, which was assigned to the (202) facet of χ-Fe_5_C_2_ (Supplementary Fig. 17e). The exposed χ-Fe_5_C_2_ facets of Fe_3_O_4_@χ-Fe_5_C_2_ nanocubes were preserved after 100 h on stream. The EELS image implied that the core region mainly comprised Fe and O elements while the shell region contained Fe and C elements (Supplementary Fig. 17f ). We also conducted Mössbauer spectroscopy to characterize the compositions of the iron phase in the used Fe_3_O_4_@χ-Fe_5_C_2_ nanocubes after 100 h on stream (Supplementary Fig. 18a). The content of χ-Fe_5_C_2_ increased from 33.2% to 39.8% after the reaction (Supplementary Table 4).

In contrast, the CO conversion of Fe_3_O_4_@χ-Fe_5_C_2_ octahedra/SiC declined continuously. The conversion was only 11.7% after 100 h, about half of that (20.1%) at the beginning (Fig. 4b). To investigate the reason for the deactivation, we characterized Fe_3_O_4_@χ-Fe_5_C_2_ octahedra/SiC after 100 h on stream. As shown in Supplementary Fig. 19a, b, the solid octahedra collapsed after the reaction. The formation of Fe_3_O_4_@χ-Fe_5_C_2_ octahedra with multiple voids was ascribed to the Kirkendall effect^37–39^. Specifically, as the blockage of active sites by long-chain hydrocarbons and non-graphitic carbon, Fe_3_O_4_@χ-Fe_5_C_2_ octahedra were gradually deactivated. Meanwhile, the carbon chemical potential of reaction conditions changed. The dynamic balance of the hydrocarbon production, surface oxidation, and carburization in the syngas environment was broken. This led to the diffusion of Fe atoms between the Fe_3_O_4_ core and χ-Fe_5_C_2_ shell. The void formation in Fe_3_O_4_@χ-Fe_5_C_2_ octahedra due to differential diffusion rates of Fe atoms. The thickness of the shell increased from 1.7 nm to 3.5 nm after the reaction (Fig. 3c and Supplementary Fig. 19c). The lattice parameters of the inner core and the outer shell were measured as 0.48 nm and 0.21 nm, which were assigned to the (111) facet of Fe_3_O_4_ and (112) facet of χ-Fe_5_C_2_, respectively (Supplementary Fig. 19d, e). The exposed χ-Fe_5_C facet of Fe_3_O_4_@χ-Fe_5_C_2_ octahedra was preserved after the reaction. The distribution of Fe_3_O_4_ at the core and χ-Fe_5_C_2_ at the shell was supported by the EELS image (Supplementary Fig. 19f). The content of χ-Fe_5_C_2_ in Fe_3_O_4_@χ-Fe_5_C_2_ octahedra increased from 29.5% to 40.4% after 100 h on stream (Supplementary Fig. 18b and Supplementary Table 4). The thickness of the shell layer for both Fe_3_O_4_@χ-Fe_5_C_2_ nanocubes and octahedra increased after the reaction. Therefore, the stabilities of Fe_3_O_4_@χ-Fe_5_C_2_ nanocrystals/SiC were also affected by the exposed facets.

To investigate the textural properties, we carried out N_2_ physisorption characterizations. The pore-diameter distributions were analyzed using the Barrett-Joyner-Halenda (BJH) method. As shown in (Supplementary Fig. 4c, d), the surface layer of both Fe_3_O_4_@χ-Fe_5_C_2_ nanocubes and octahedra were not porous. The absence of pores at the surface layer was also confirmed by HAADF-STEM images (Figs. 1b and 3b). We also measured the textural properties of spent Fe_3_O_4_@χ-Fe_5_C_2_ nanocubes and octahedra after 100 h on stream (Supplementary Fig. 20a, b). The surface layer of spent Fe_3_O_4_@χ-Fe_5_C_2_ nanocubes remained nonporous after 100 h on stream (Supplementary Fig. 20c). In contrast, spent Fe_3_O_4_@χ-Fe_5_C_2_ octahedra contained mesopores as revealed by the pore-diameter distribution and HAADF-STEM image (Supplementary Fig. 20d). The average mesopore diameter was determined as 16.0 nm by the BJH method (Supplementary Fig. 20d).

To investigate other underlying mechanisms for catalyst deactivation, we employed Raman spectroscopy to analyze the surfaces of Fe_3_O_4_@χ-Fe_5_C_2_ nanocubes and octahedra after 100 h. The presence of peaks at 1330 cm^−1^ indicated the existence of disordered carbon (D band), while those at 1592 cm^−1^ signified graphite (G band) (Supplementary Fig. 21a, b). Significantly higher intensities of these peaks were observed on the surface of spent Fe_3_O_4_@χ-Fe_5_C_2_ octahedra compared with spent nanocubes, suggesting a greater accumulation of deposited carbon on the octahedral surface (Supplementary Fig. 21a, b). For a more precise comparison, we conducted thermogravimetric analysis (TGA) under the N_2_ atmosphere on both Fe_3_O_4_@χ-Fe_5_C_2_ nanocubes and octahedra after the reaction. The weight loss between 200 and 500 °C was attributed to the removal of long-chain hydrocarbons from the surface, while the weight loss beyond 500 °C was associated with the loss of non-graphitic carbon. In the case of spent Fe_3_O_4_@χ-Fe_5_C_2_ nanocubes, a weight loss of 6.2 wt% was observed (Supplementary Fig. 21c). Conversely, during TGA testing, spent Fe_3_O_4_@χ-Fe_5_C_2_ octahedra exhibited a total weight loss of 13.5 wt% due to long-chain hydrocarbons and deposited carbon (Supplementary Fig. 21d). The higher residual weight of long-chain hydrocarbons and deposited carbon on the octahedral structure suggests a more pronounced blockage of active sites compared to nanocubes. Hence, we hypothesize that carbon deposition also contributes to the deactivation of Fe_3_O_4_@χ-Fe_5_C_2_ octahedra.

### Mechanistic insights into the facet effect

To rationalize the facet-dependent FTS activity, we explored CO dissociation pathways by conducting in-situ diffuse reflection infrared spectroscopy (DRIFTS) measurements at 270 °C. When Fe_3_O_4_@χ-Fe_5_C_2_ nanocubes were exposed to 1 bar of CO and purged with He, three sets of peaks were observed (Fig. 4c). The peaks at 2173 and 2115 cm^−1^ were assigned to the gaseous CO, while the peak at 2058 cm^−1^ arose from the stretching vibration of linearly adsorbed CO^19,40–42^. The peaks at 2360 and 2336 cm^−1^ corresponded to the gaseous CO_2_, indicating the direct dissociation of CO on Fe_3_O_4_@χ-Fe_5_C_2_ nanocubes. As for the exposure to CO and purging with H_2_, other sets of peaks emerged besides the peaks for CO and gaseous CO_2_ (Fig. 4c). Specifically, the peak at 2955 cm^−1^ derived from the asymmetrical stretching vibration of C-H in CH_3_* (refs. ^43,44^). The peaks at 2919 and 2849 cm^−1^ were indexed as the asymmetrical and symmetrical stretching vibrations of C-H in CHO* and CH_2_*, respectively^45,46^. Notably, the peak at 1741 cm^−1^ was ascribed to the stretching vibration of C = O in CHO* species, which indicated the existence of a hydrogen-assisted dissociation route^47,48^. For Fe_3_O_4_@χ-Fe_5_C_2_ octahedra, when the sample was exposed to CO and purged with He, the peaks for gaseous CO_2_ appeared (Fig. 4d). As for the treatment with CO and purging with H_2_, the in-situ DRIFTS profile showed the peaks for CH_3_*, CH_2_*, CO_2_, and CO, in the absence of CHO* or COH* (Fig. 4d). We also conducted in-situ DRIFTS experiments under 20 bar of syngas, simulating realistic reaction environments (Supplementary Fig. 22, Supplementary Table 5). The appearance of gaseous CO_2_ peaks at 2360 and 2336 cm^−1^ provided evidence for direct dissociation occurring on both Fe_3_O_4_@χ-Fe_5_C_2_ nanocubes and octahedra (Supplementary Fig. 22, Supplementary Table 5). Moreover, the presence of CHO* species, as indicated by a peak at 1741 cm^−1^, was observed solely on the Fe_3_O_4_@χ-Fe_5_C_2_ nanocubes catalyst and absent on the octahedral counterpart (Supplementary Fig. 22, Supplementary Table 5). Therefore, Fe_3_O_4_@χ-Fe_5_C_2_ octahedra enabled the direct dissociation of CO, while both direct and hydrogen-assisted CO dissociation routes existed on Fe_3_O_4_@χ-Fe_5_C_2_ nanocubes (Supplementary Fig. 23).

To investigate the activation energy of different CO dissociation routes, we plotted the area of linearly adsorbed CO peak at 2058 cm^−1^ as a function of time at 190, 230, and 270 °C (Supplementary Figs. 24–27). Based on the slope of the decrease in peak area with purge time, the rate of CO dissociation (*k*) was obtained. Activation energies of CO dissociation were calculated with the Arrhenius equation^7^. For Fe_3_O_4_@χ-Fe_5_C_2_ nanocubes, the activation energy for hydrogen-assisted dissociation of CO was 68.6 kJ mol^−1^, significantly lower than that (103.7 kJ mol^−1^) direct dissociation of CO (Supplementary Fig. 28a). With regard to Fe_3_O_4_@χ-Fe_5_C_2_ octahedra, the activation energy (83.2 kJ mol^−1^) of CO dissociation with H_2_ approximated to that (80.4 kJ mol^−1^) without H_2_ (Supplementary Fig. 28b). Therefore, Fe_3_O_4_@χ-Fe_5_C_2_ nanocrystals exhibited facet-dependent FTS activities derived from the alteration of reaction paths which changed the activation energy. Specially, Fe_3_O_4_@χ-Fe_5_C_2_ nanocubes followed hydrogen-assisted CO dissociation which lowered the activation energy, relative to that of direct CO dissociation over Fe_3_O_4_@χ-Fe_5_C_2_ octahedra.

We conducted DFT calculations to rationalize CO direct dissociation route and hydrogen-assisted dissociation path on χ-Fe_5_C_2_(202) and χ-Fe_5_C_2_(112) surface. For χ-Fe_5_C_2_(202) surface, the hydrogen-assisted CO dissociation route is the dominating route, since its energy barrier of the rate-limiting step (CO* + H* → HCO*) 1.23 eV is much lower than the direct CO dissociation route with an energy barrier as high as 2.85 eV (Supplementary Figs. 29–32). For χ-Fe_5_C_2_(112) surface, the direct CO dissociation route exhibited a lower energy barrier (1.37 eV) than the hydrogen-assisted CO dissociation route (1.54 eV), implying the direct dissociation as the main route of CO dissociation (Supplementary Figs. 33–36). Besides, the energy barrier of the hydrogen-assisted CO dissociation route on the χ-Fe_5_C_2_(202) surface is lower than that of the CO direct dissociation on the χ-Fe_5_C_2_(112) surface (Supplementary Figs. 32 and 35). The DFT conclusion was consistent with in-situ DRIFTS spectra result.

We explored the adsorption of CO and H_2_ by conducting pulse chemisorption measurements to rationalize why Fe_3_O_4_@χ-Fe_5_C_2_ nanocubes/SiC exhibited better carbon-chain growth ability. The amount of adsorbed gas was calculated on the difference between the total amount of gas injected and the amount measured at the outlet from the sample. The amount of adsorbed CO over Fe_3_O_4_@χ-Fe_5_C_2_ nanocubes/SiC was 24.1 µmol g^−1^, higher than that (19.5 µmol g^−1^) of Fe_3_O_4_@χ-Fe_5_C_2_ octahedra (Supplementary Fig. 12). While the amount of adsorbed H_2_ over Fe_3_O_4_@χ-Fe_5_C_2_ nanocubes/SiC was 7.3 µmol g^−1^, lower than that (11.0 µmol g^−1^) of the octahedral counterpart (Supplementary Fig. 37). The results indicated that Fe_3_O_4_@χ-Fe_5_C_2_ nanocubes/SiC improved the CO adsorption and suppressed the H_2_ adsorption compared to the octahedral counterpart. Thus, the surface CO/H_2_ ratio of Fe_3_O_4_@χ-Fe_5_C_2_ nanocubes/SiC was 3.3, higher than that (1.8) of the octahedral counterpart (Supplementary Fig. 38). With the increase of the surface CO/H_2_ ratio, the CH_4_ production over Fe_3_O_4_@χ-Fe_5_C_2_ nanocubes/SiC was suppressed, while hydrocarbon products shifted to long-chain hydrocarbons. We also conducted DFT calculations to investigate the facet effect on CH_4_ production. The CH_2_* + H* energy barrier of χ-Fe_5_C_2_(202) is 1.03 eV, higher than that (0.70 eV) of χ-Fe_5_C_2_(112) (Supplementary Figs. 39–41). The energy barrier of the CH_2_* + CH_2_* step over χ-Fe_5_C_2_(202) facet is 0.70 eV, lower than that (1.03 eV) of CH_2_* + H*. As for χ-Fe_5_C_2_(112) facet, the energy barrier of the CH_2_* + CH_2_* step is 0.60 eV, approaching to that (0.7 eV) of CH_2_* + H*. Thus, compared with Fe_3_O_4_@χ-Fe_5_C_2_ nanocubes, the octahedral counterpart benefits the CH_4_ production.

We demonstrated the dependence of catalytic performance on the exposed facet of iron carbides in FTS. Uniformly exposed {202} and {112} facets of χ-Fe_5_C_2_ were successfully fabricated as the model system. With the help of well-defined catalysts, we identified the intrinsically active, selective, and stable facets of χ-Fe_5_C_2_. Our findings deepen the understanding of Fe-based FTS catalysts from the phase and size to the facet. In addition, this work also provides a facile method to precisely control the exposed surfaces of iron carbides for both future fundamental studies and practical applications.

## Methods

### Chemicals and materials

Oleylamine (OAm, >70%), oleic acid (OA, 90%), benzyl ether (BE, 99%), iron(III) acetylacetonate (Fe(acac)_3_, >99.9%), and 4-biphenylcarboxylic acid (99%) were obtained from Sigma-Aldrich. SiC (β-phase, 99.8%) was obtained from Adamas-beta®. All other chemicals were analytical grade and purchased from Sinopharm Chemical Reagent Co., Ltd. Deionized water with a resistivity of 18.2 MΩ cm was used for the preparation of all aqueous solutions (Milli-Q®).

### Synthesis of Fe_3_O_4_ nanocrystals

In a typical synthesis of Fe_3_O_4_ nanocubes, Fe(acac)_3_ (1.4 g) and 4-biphenylcarboxylic acid (1.0 g) were dissolved in a mixture of BE (20.0 mL) and OA (2.5 mL). The solution was degassed at 120 °C for 30 min. Subsequently, the solution was heated to 290 °C at 10 °C min^−1^ and kept for 30 min with a stirring rate of 300 rpm. As for the synthesis of Fe_3_O_4_ octahedra, Fe(acac)_3_ (1.0 g) was dissolved in a mixture of BE (20.0 mL), OAm (2.3 mL), and OA (1.6 mL), followed by degassing at 120 °C for 30 min. The solution was heated to 220 °C at 20 °C min^−1^ with a stirring rate of 300 rpm and kept for 1 h. Afterward, the solution was heated to 300 °C at 20 °C min^−1^ and kept for 2 h. After the solution had been cooled down to room temperature, the products were precipitated by ethanol, washed three times with hexane, and re-dispersed in hexane.

### Synthesis of Fe_3_O_4_@χ-Fe_5_C_2_ nanocrystals

For the synthesis of Fe_3_O_4_@χ-Fe_5_C_2_ nanocrystals, as-prepared Fe_3_O_4_ nanocrystals (50 mg) were loaded into a fixed-bed reactor with an inner diameter of 9 mm. Fe_3_O_4_ nanocrystals were reduced in H_2_ under 1 bar with a gas-flow rate of 100 mL min^−1^ at 270 °C for 10 h, while the heating ramp was 1 °C min^−1^. Afterwards, the obtained samples underwent surface reconstruction under 20 bar of syngas (32 vol% H_2_, 64 vol% CO, and 4 vol% Ar) with a space velocity of 2400 mL h^−1^ g_cat_^−1^ at 270 °C for 20 h. Considering that the iron carbides were highly sensitive to the atmosphere, we used inert gas to protect and store the catalyst after reaction. Specifically, we switched the feed gas to N_2_ when the reaction was stopped. The reaction tubes were sealed via valves at both ends after cooling the reactor to room temperature. Afterwards, the reaction tubes were transferred to a N_2_-filled glove box. The samples were stored in the glove box before characterizations.

### Synthesis of pure χ-Fe_5_C_2_ nanocrystals

Octadecylamine of 14.5 g and cetyl trimethyl ammonium bromide (CTAB) of 0.113 g were mixed in a four-neck flask under stirring and degassed under the N_2_ flow, followed by being heated to 120 °C. Afterwards, Fe(CO)_5_ (3.6 mmol) was injected into the mixture under the N_2_ blanket. The mixture was heated to 180 °C at 10 °C min^−1^ and kept at this temperature for 10 min. Subsequently, the mixture was further heated to 350 °C at 10 °C min^−1^ and kept at this temperature for 10 min. After being cooled to room temperature, the products were washed with a mixture of ethanol and hexane.

### Mössbauer measurements

^57^Fe Mössbauer spectra were carried out on a Topologic 500 A spectrometer driving with a proportional counter at room temperature. The radioactive source was ^57^Co (Rh) moving in a constant acceleration mode. Data analyses were performed assuming a Lorentzian lineshape for computer folding and fitting.

### Catalytic tests

The Fischer-Tropsch reaction was carried out in a fixed-bed reactor under 20 bar of syngas at 270 °C. Generally, Fe_3_O_4_@χ-Fe_5_C_2_ nanocrystals (50 mg, 20–40 meshes) were diluted with SiC (450 mg, 20–40 meshes). The sample was loaded into a fixed-bed reactor with an inner diameter of 9 mm. Subsequently, a mixture including 96 vol% of H_2_/CO mixed gas (64 vol% H_2_, 32 vol% CO) and 4 vol% of Ar (as an internal standard) was introduced to the reactor as the feeding gas at a space velocity of 2400 mL h^−1^ g_cat_^−1^.

The gaseous products were monitored by online gas chromatographs (Shimadzu GC-2014). An ice trap with 2.0 g of solvent (*n*-dodecane) was employed to capture the liquid hydrocarbons in the effluent. The liquid products with 0.1 g of an internal standard (decalin) were analyzed using an offline Shimadzu GC-2014.

CO conversion was calculated as follows:

3 $${{{\rm{CO\; conversion}}}}=\frac{{{{{\rm{CO}}}}}_{{{{\rm{inlet}}}}}-{{{{\rm{CO}}}}}_{{{{\rm{outlet}}}}}}{{{{{\rm{CO}}}}}_{{{{\rm{inlet}}}}}}\times 100\%$$

where CO_inlet_ and CO_outlet_ are moles of CO at the inlet and outlet, respectively.

CO_2_ selectivity was calculated according to:

4 $${{{{\rm{CO}}}}}_{2}\, {{{\rm{selectivity}}}}=\frac{{{{{\rm{CO}}}}}_{{{{\rm{outlet}}}}}}{{{{{\rm{CO}}}}}_{{{{\rm{inlet}}}}}-{{{{\rm{CO}}}}}_{{{{\rm{outlet}}}}}}\times 100\%$$

where CO_2 outlet_ refers to moles of CO_2_ at the outlet.

The selectivity of hydrocarbon C_n_H_m_ was obtained according to:

5 $${{{{\rm{C}}}}}_{{{{\rm{n}}}}}{{{{\rm{H}}}}}_{{{{\rm{m}}}}}\, {{{\rm{selectivity}}}}=\frac{{{{{\rm{nC}}}}}_{n}{{{{\rm{H}}}}}_{{{{\rm{m}}}}\, {{{\rm{outlet}}}}}}{{\sum}_{{{{\rm{i}}}}}{{{\rm{i}}}}{{{{\rm{C}}}}}_{{{{\rm{i}}}}}{{{{\rm{H}}}}}_{{{{\rm{m\; outlet}}}}}}\times 100\%$$

where C_n_H_m outlet_ represents moles of individual hydrocarbon product at the outlet.

Carbon balance was calculated according to:

6 $${{{\rm{Carbon}}}\; {{\rm{balance}}}}=\frac{{{\sum}_{{{{\rm{i}}}}}{{{\rm{i}}}}{{{{\rm{C}}}}}_{{{{\rm{i}}}}}{{{{\rm{H}}}}}_{{{{\rm{m}}}\; {{\rm{outlet}}}}}}+{{{{\rm{CO}}}}}_{2}}{{{{{\rm{CO}}}}}_{{{{\rm{inlet}}}}}-{{{{\rm{CO}}}}}_{{{{\rm{outlet}}}}}}\times 100\%$$

where C_n_H_m_ and CO_2_ represent the moles of the produced hydrocarbons and CO_2_, respectively. CO_inlet_ and CO_outlet_ are moles of CO at the inlet and outlet, respectively. The carbon balances were over 95.0%.

### In-situ DRIFTS measurements after different gas treatments

In-situ DRIFTS experiments were conducted in an elevated-pressure cell (DiffusIR Accessory PN 041-10XX) with a Fourier transform infrared spectrometer (TENSOR II Sample Compartment RT-DLaTGS) and a liquid-nitrogen-cooled MCT detector. The outlet gas was analyzed by a mass spectrometer (Hiden HPR20). Spectra were measured accumulating 64 scans at a resolution of 4 cm^−1^. Prior to the test, the samples were cleaned in He with a gas-flow rate of 100 mL min^−1^ at 270 °C for 1 h. Then the samples were exposed to 1 bar of CO for 30 min and subsequently purged by He or H_2_ for 30 min at 270 °C. As for the in-situ DRIFTS measurements at different temperatures, the background spectra of the samples were acquired under He after the temperature of the samples dropped to a specified temperature. Then, the samples were exposed to 1 bar of CO for 30 min and subsequently purged with He or H_2_ at a specified temperature. As for the in-situ DRIFTS experiments under 20 bar of syngas, thorough cleaning of the samples in He at 270 °C for 1 h ensured the elimination of any contaminants. Background spectra were acquired under He flow, followed by exposure to a 20-bar syngas mixture (CO/H_2_) at 270 °C for 30 min.

### DFT methods

Spin-polarized DFT calculations were conducted using the Vienna ab initio simulation package (VASP)^49^. The projector-augmented wave method and the Perdew-Burke-Ernzerhof functional were implemented in the code^50^. Fe_5_C_2_(202) and Fe_5_C_2_(112) slab were established with the same stoichiometric atoms (Fe_60_C_24_). A plane-wave basis set with a cutoff energy of 400 eV was used with the K-points of 3 × 3 × 1 and vacuum thickness of 15 Å. During the structure optimization, the two layers of atoms at the bottom were fixed, while the others include the adsorbates were relaxed with the electronic convergence of 0.02 eV/Å. For each elementary step, the initial states and final states are firstly optimized. The transition states are searched using the climbing image nudged elastic band method (CI-NEB) and confirmed by the vibrational frequencies analysis^51^.

### CO pulse chemisorption measurements

CO pulse chemisorption measurements were performed using a Micromeritics Autochem 2920 chemisorption analyzer with an active loop volume of 0.1 mL. In a typical measurement, 100 mg of Fe_3_O_4_@χ-Fe_5_C_2_ nanocrystals/SiC were packed into a reactor with a quartz tube. Prior to the test, the samples were cleaned in He with a gas-flow rate of 100 mL min^−1^ at 270 °C for 5 h. After cooling down to 50 °C under He flow, CO/He pulses (10 vol% CO and 90 vol% He) were injected until adsorption reached saturation. The amount of adsorbed CO was calculated on the difference between the total amount of CO injected and the amount measured at the outlet from the sample. The metal dispersion was calculated by assuming the ratio of CO to surface metal atom as 1:1.

TOF number was calculated according to:

7 $${{{\rm{TOF}}}}=	{{{\rm{CO}}}}\; {{{\rm{conversion}}}}{{\times }}{{{\rm{moles}}}}\; {{{\rm{of}}}}\; {{{\rm{CO}}}}\; {{{\rm{in}}}}\; {{{\rm{syngas}}}}{{\times }}{{{\rm{gas}}}}\\ 	-{{{\rm{flow}}}}\; {{{\rm{rate}}}}\div{{{\rm{moles}}}}\; {{{\rm{of}}}}\; {{{\rm{surface}}}}\; {{{\rm{Fe}}}}\; {{{\rm{atoms}}}}$$

### H_2_ pulse chemisorption measurements

H_2_ pulse chemisorption measurements were performed using a Micromeritics Autochem 2920 chemisorption analyzer with an active loop volume of 0.1 mL. In a typical measurement, 100 mg of Fe_3_O_4_@χ-Fe_5_C_2_ nanocrystals/SiC were packed into a reactor with a quartz tube. Prior to the test, the samples were cleaned in He with a gas-flow rate of 100 mL min^−1^ at 270 °C for 5 h. After cooling down to 50 °C under He flow, H_2_/Ar pulses (10 vol% H_2_ and 90 vol% Ar) were injected until adsorption reached saturation. The amount of adsorbed H_2_ was calculated on the difference between the total amount of H_2_ injected and the amount measured at the outlet from the sample.

### Instrumentations

TEM images were taken using a Hitachi H-7700 transmission electron microscope at an acceleration voltage of 100 kV. HAADF and EDS analysis were collected on a JEOL ARM-200F field-emission transmission electron microscope operating at 200 kV accelerating voltage. XPS measurements were conducted on an ESCALAB 250 (Thermo-VG Scientific, USA) with an Al Kα X-ray source (1486.6 eV protons) in Constant Analyzer Energy (CAE) mode with a pass energy of 30 eV for all spectra. XRD characterization was performed using a Philips X’Pert Pro X-ray diffractometer with a monochromatized Cu Kα radiation source and a wavelength of 0.1542 nm. BET measurements were carried out on Micromeritics AutoChem II 2020. TGA spectra were conducted on Pyris Diamond TG-DTG. Raman spectra were detected by a Renishaw RM3000 Micro-Raman system with a 514.5 nm Ar laser.

### Supplementary information

Supplementary information

Peer Review File

## Data Availability

The data generated in this study are provided in the Supplementary Information.
