# Supplementary material for: Facet sensitivity of iron carbides in Fischer-Tropsch synthesis
Source: Nat Commun. 2024 Jul 19;15:6108. doi: 10.1038/s41467-024-50544-1 (PMC11271519; doi:10.1038/s41467-024-50544-1)
Supplement: Supplementary file 1 — Supplementary information [file 41467_2024_50544_MOESM1_ESM.doc]

Supplementary information for

**Facet sensitivity of iron carbides in Fischer-Tropsch synthesis**

Wenlong Wu^1,2,3†^, Jiahua Luo^2†^, Jiankang Zhao^2^, Menglin Wang^2^, Lei Luo^2^, Sunpei Hu^2^, Bingxuan He^2^, Chao Ma^4^, Hongliang Li^2,3*^, Jie Zeng^1,2*^

^1^School of Chemistry & Chemical Engineering, Anhui University of Technology, Ma’anshan, Anhui 243002, P. R. China

^2^Hefei National Research Center for Physical Sciences at the Microscale, Key Laboratory of Strongly-Coupled Quantum Matter Physics of Chinese Academy of Sciences, Key Laboratory of Surface and Interface Chemistry and Energy Catalysis of Anhui Higher Education Institutes, Department of Chemical Physics, University of Science and Technology of China, Hefei, Anhui 230026, P. R. China

^3^National Synchrotron Radiation Laboratory, University of Science and Technology of China, Hefei, Anhui 230026, P. R. China

^4^College of Materials Science and Engineering, Hunan University, Changsha, 410082, P. R. China

^†^These authors contributed equally to this work.

^*^e-mail: lihl@ustc.edu.cn; zengj@ustc.edu.cn


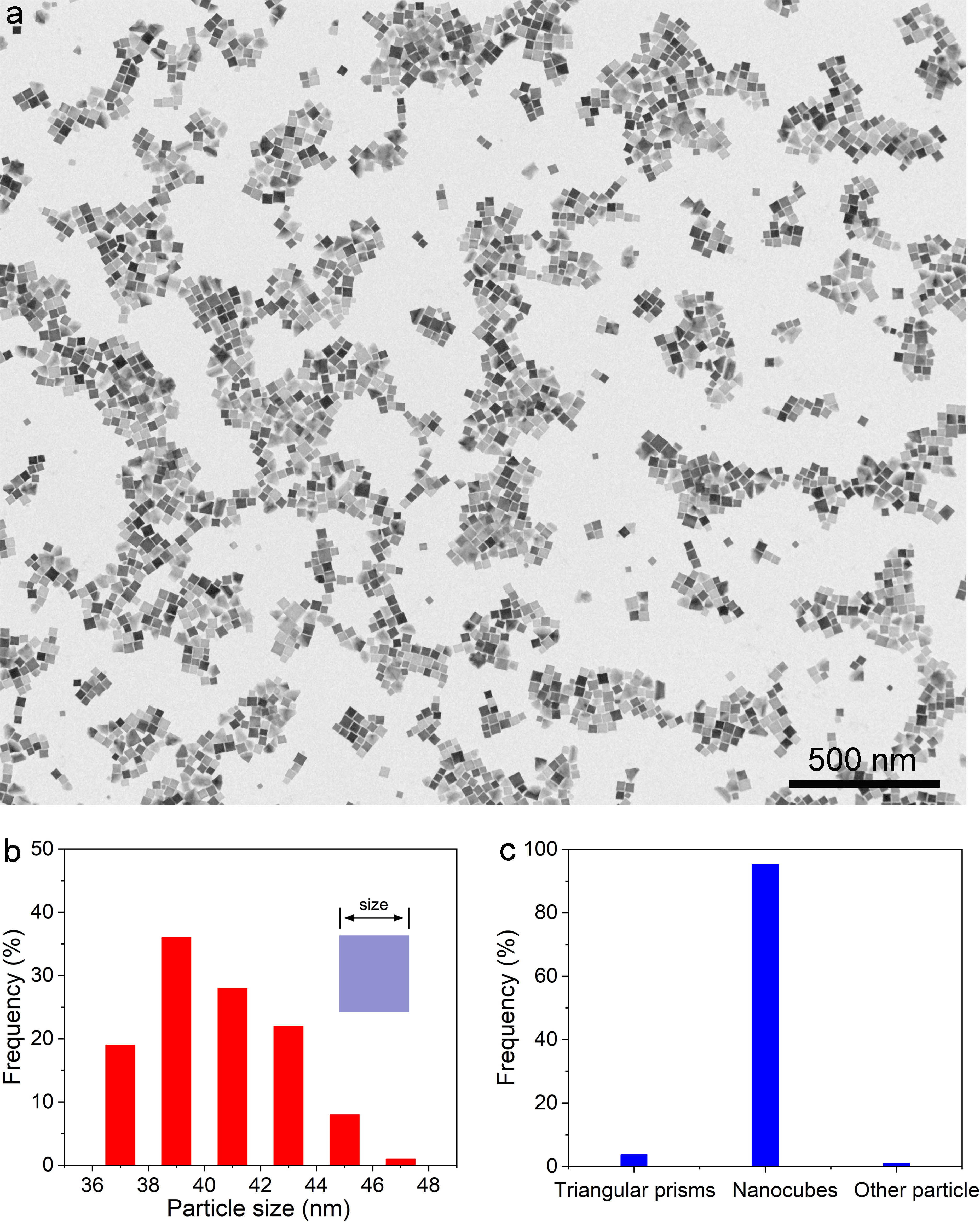


**Supplementary Figure 1 | Structural characterizations of Fe_3_O_4_ nanocubes**. (**a**) TEM image of Fe_3_O_4_ nanocubes. (**b**) Size distribution of the corresponding Fe_3_O_4_ nanocubes. The average particle size was 40.5±3.9 nm. (**c**) Contents of nanocrystals with different shapes. The purity of Fe_3_O_4_ nanocubes was 95.3%.


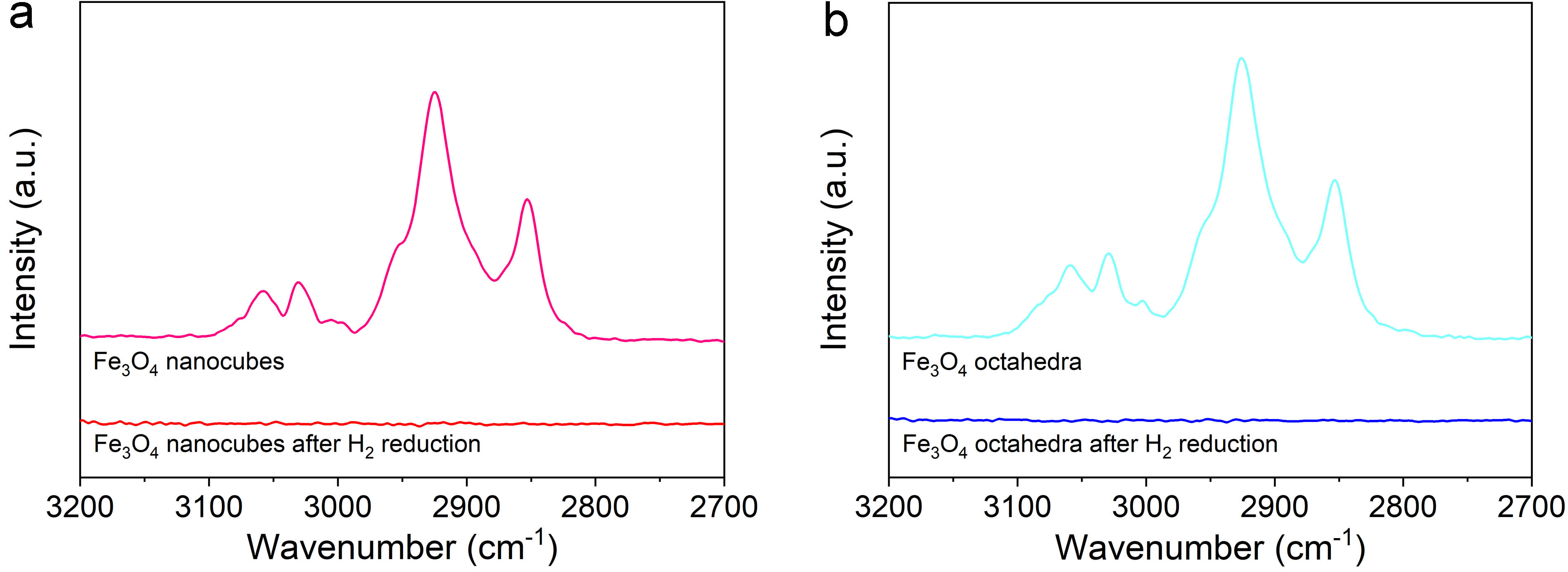


**Supplementary Figure 2 | FTIR spectra.** (**a**) FTIR spectra of Fe_3_O_4_ nanocubes before/after H_2_ reduction. (**b**) FTIR spectra of Fe_3_O_4_ octahedra before/after H_2_ reduction.


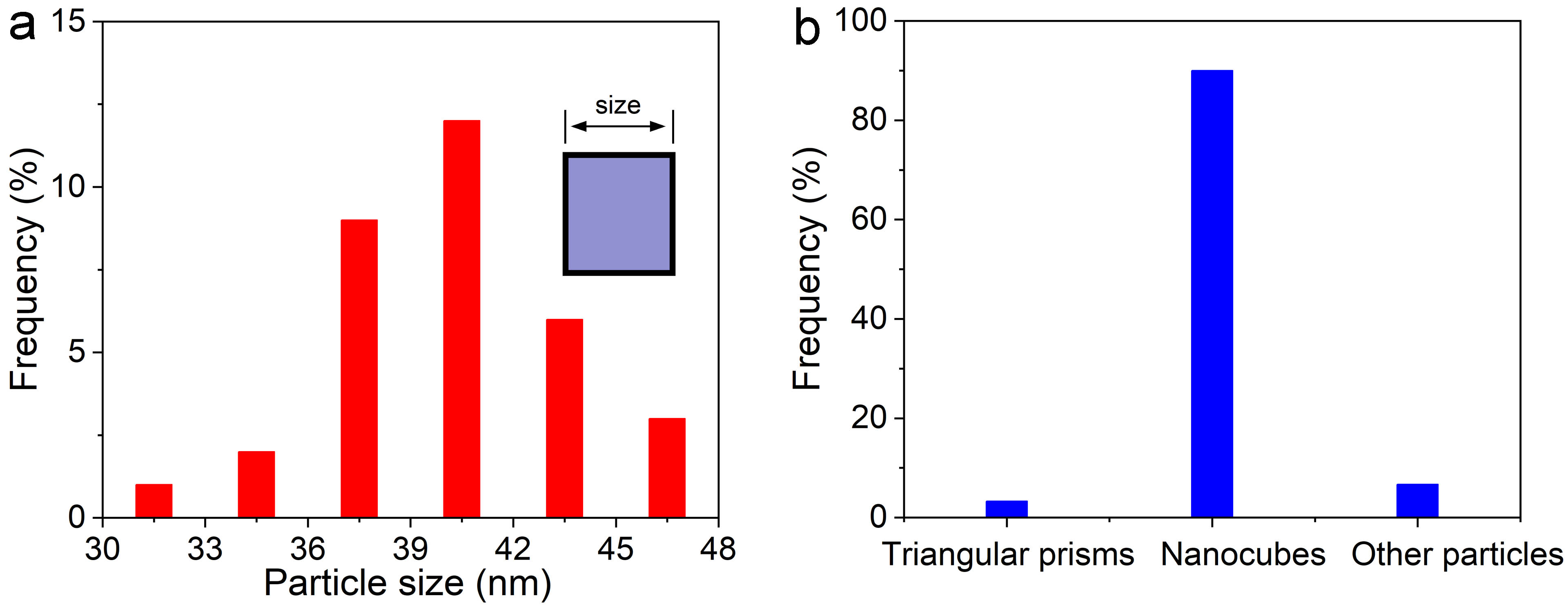


**Supplementary Figure 3 | Statistics of Fe_3_O_4_@χ-Fe_5_C_2_ nanocubes from Figure 1a.** (**a**) Size distribution of Fe_3_O_4_@χ-Fe_5_C_2_ nanocubes. The average particle size was 40.4±3.8 nm. (**b**) Contents of nanocrystals with different shapes. The purity of Fe_3_O_4_@χ-Fe_5_C_2_ nanocubes was 90.0%.


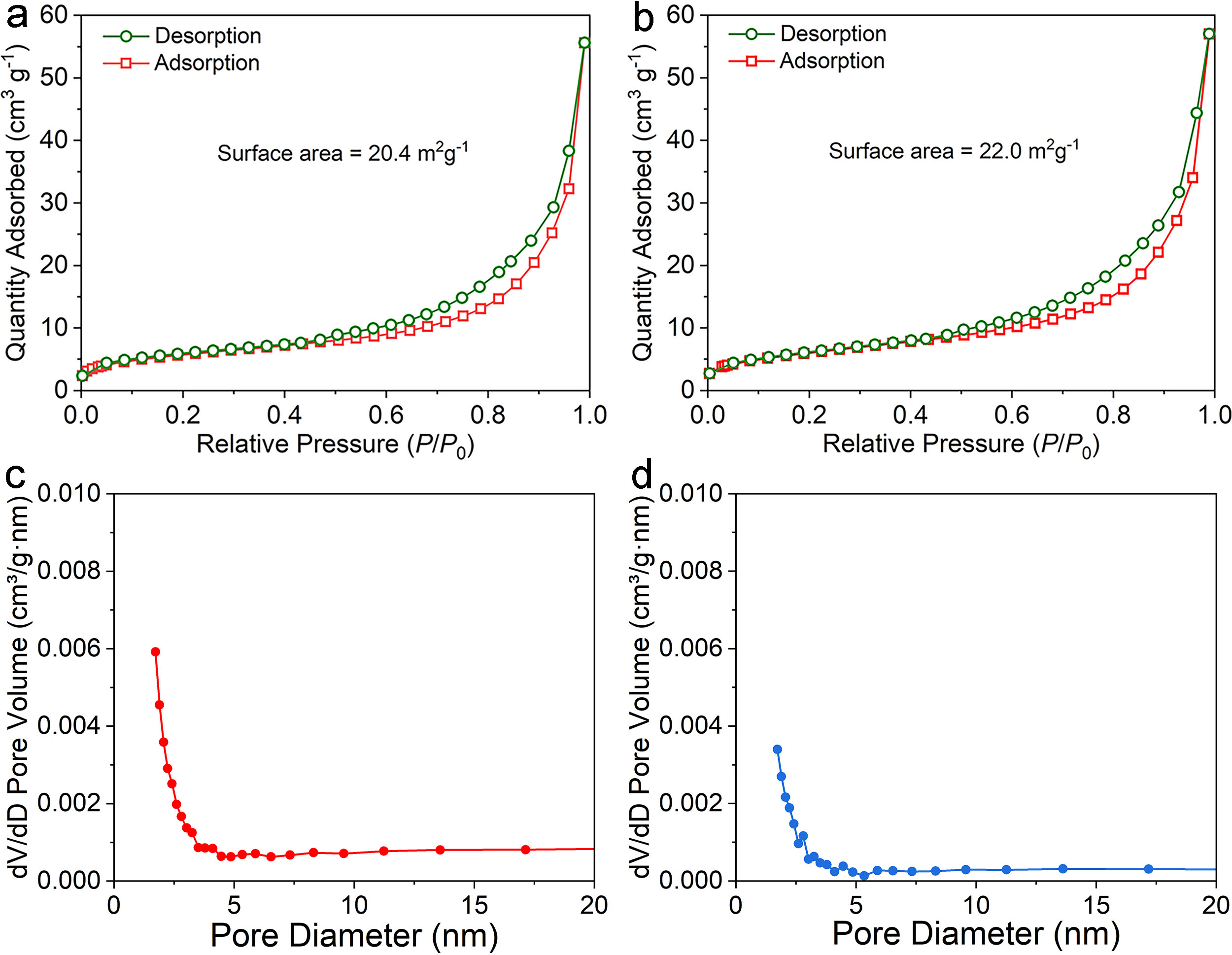


**Supplementary Figure 4 | Textural properties of Fe_3_O_4_@χ-Fe_5_C_2_ nanocubes and octahedra before reaction.** (**a, b**) Nitrogen adsorption and desorption isotherm of (**a**) Fe_3_O_4_@χ-Fe_5_C_2_ nanocubes and (**b**) Fe_3_O_4_@χ-Fe_5_C_2_ octahedra. (**c, d**) Pore-size distributions of (**c**) Fe_3_O_4_@χ-Fe_5_C_2_ nanocubes and (**d**) Fe_3_O_4_@χ-Fe_5_C_2_ octahedra derived from the nitrogen adsorption-desorption isotherms by the BJH method.


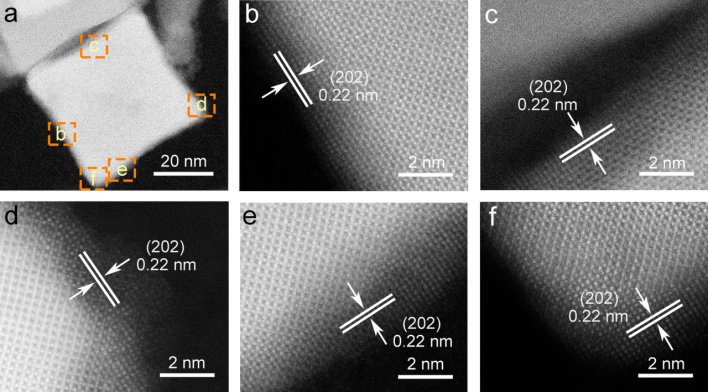


**Supplementary Figure 5 | Structural characterizations of the Fe_3_O_4_@χ-Fe_5_C_2_ nanocube.** (**a**) HAADF-STEM image of an individual Fe_3_O_4_@χ-Fe_5_C_2_ nanocube. (**b-f**) Magnified HAADF-STEM images of the region marked by the corresponding boxes in panel **a**.

**Supplementary Table 1 | Mössbauer parameters of Fe_3_O_4_@χ-Fe_5_C_2_ nanocubes and Fe_3_O_4_@χ-Fe_5_C_2_ octahedra**. The isomer shift (IS), quadrupole splitting (QS), hyperfine field, and spectral contribution are given.

| Samples | Phase ascription | Mössbaure parameters | | | |
| --- | --- | --- | --- | --- | --- |
|  |  | IS (mm/s) | QS (mm/s) | Hyperfine field (T) | Spectral contribution (%) |
| Fe_3_O_4_@χ-Fe_5_C_2_ nanocubes | Fe_3_O_4_ (A) | 0.28 | -0.01 | 48.8 | 47.5% |
|  | Fe_3_O_4_ (B) | 0.65 | 0.01 | 45.7 | 15.9% |
|  | χ-Fe_5_C_2_ (A) | 0.22 | 0.10 | 21.9 | 11.0% |
|  | χ-Fe_5_C_2_ (B) | 0.20 | -0.01 | 18.7 | 14.5% |
|  | χ-Fe_5_C_2_ (C) | 0.19 | -0.07 | 11.1 | 7.7% |
|  | Fe(II)/Fe(III) | 0.28 | 1.06 | - | 3.4% |
| Fe_3_O_4_@χ-Fe_5_C_2_ octahedra | Fe_3_O_4_ (A) | 0.32 | -0.02 | 49.6 | 49.1% |
|  | Fe_3_O_4_ (B) | 0.65 | 0.02 | 45.8 | 16.0% |
|  | χ-Fe_5_C_2_ (A) | 0.24 | 0.10 | 21.7 | 13.5% |
|  | χ-Fe_5_C_2_ (B) | 0.19 | -0.02 | 18.1 | 10.8% |
|  | χ-Fe_5_C_2_ (C) | 0.20 | 0.06 | 11.7 | 5.2% |
|  | Fe(II)/Fe(III) | 0.41 | 1.01 | - | 5.4% |


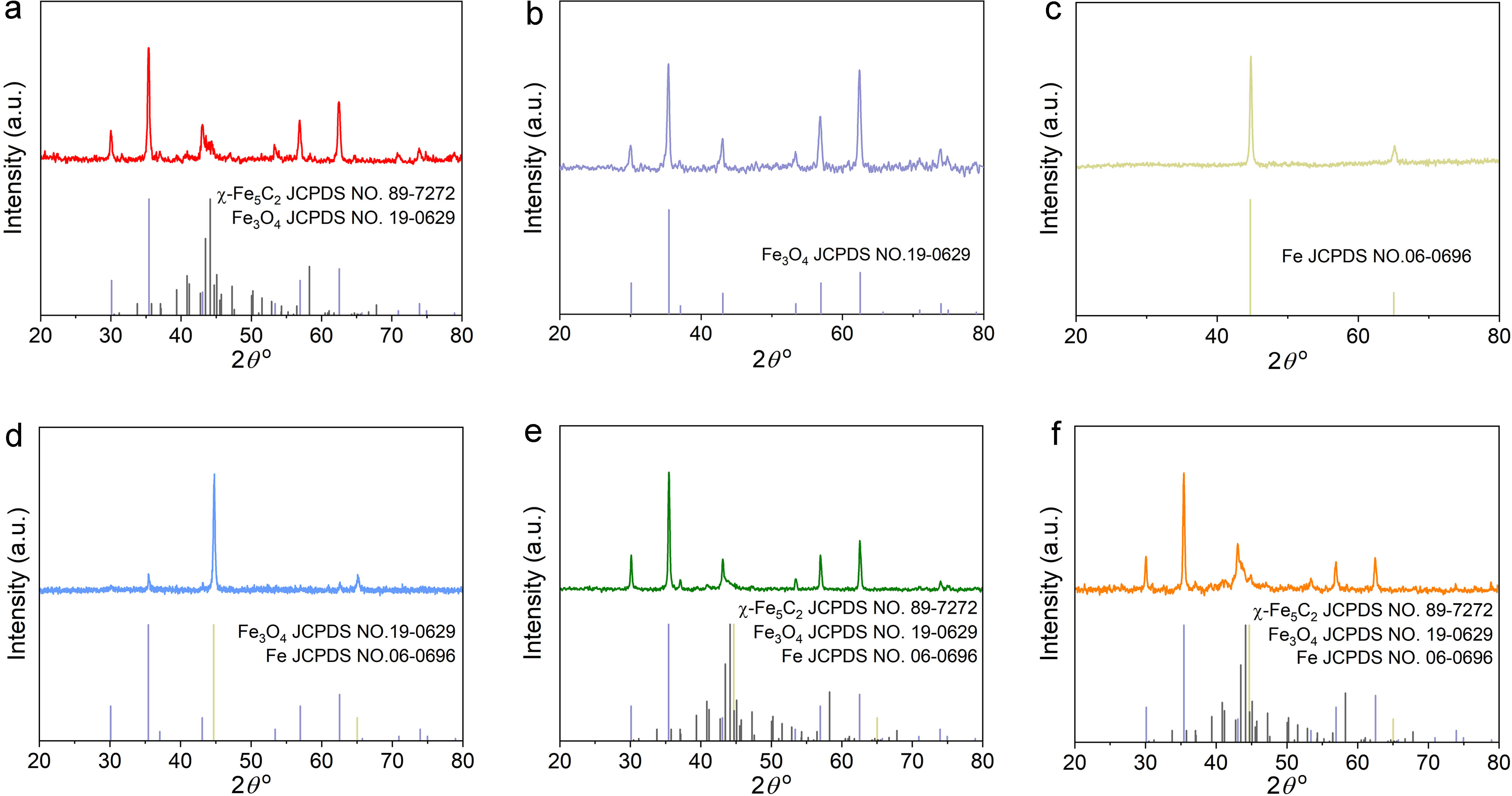


**Supplementary Figure 6 | The corresponding XRD patterns of evolution from Fe_3_O_4_ nanocubes to Fe_3_O_4_@χ-Fe_5_C_2_ nanocubes in Figure 2.** (**a**) XRD pattern of Fe_3_O_4_@χ-Fe_5_C_2_ nanocubes after the treatment of Fe nanocubes with syngas for 20 h. (**b**) XRD pattern of Fe_3_O_4_ nanocubes. (**c**) XRD pattern of Fe nanocubes. (**d-f**) XRD patterns of Fe_3_O_4_@χ-Fe_5_C_2_ nanocubes after the treatment of Fe nanocubes with syngas for 2, 5, and 10 h, respectively.

**Supplementary Table 2 | Quantitative analysis of the XRD patterns.** The calculation was based on the Ratio of Intensity Reference (RIR) method.

| sample | Iron phase | Mass fraction (wt.%) |
| --- | --- | --- |
| Fe_3_O_4_@χ-Fe_5_C_2_ nanocubes | Fe_3_O_4_ | 70.2 |
|  | χ-Fe_5_C_2_ | 29.8 |
| Fe_3_O_4_@χ-Fe_5_C_2_ octahedra | Fe_3_O_4_ | 72.4 |
|  | χ-Fe_5_C_2_ | 27.6 |


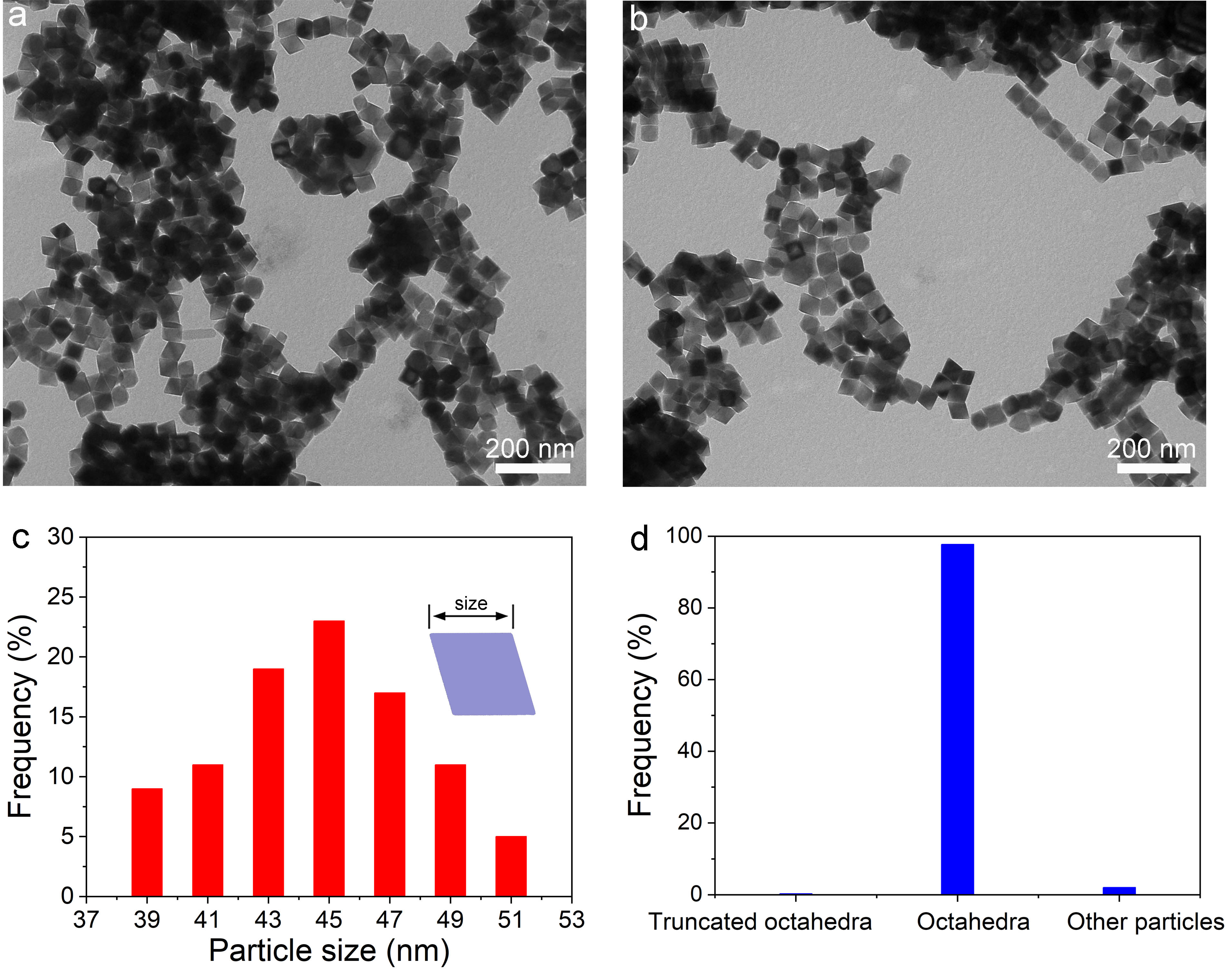


**Supplementary Figure 7 | Structural characterizations of Fe_3_O_4_ octahedra.** (**a-b**) TEM images of Fe_3_O_4_ octahedra. (**c**) Size distribution of the corresponding Fe_3_O_4_ octahedra. The average particle size was 44.8±3.3 nm. (**d**) Contents of nanocrystals with different shapes. The purity of Fe_3_O_4_ octahedra is 97.7%.


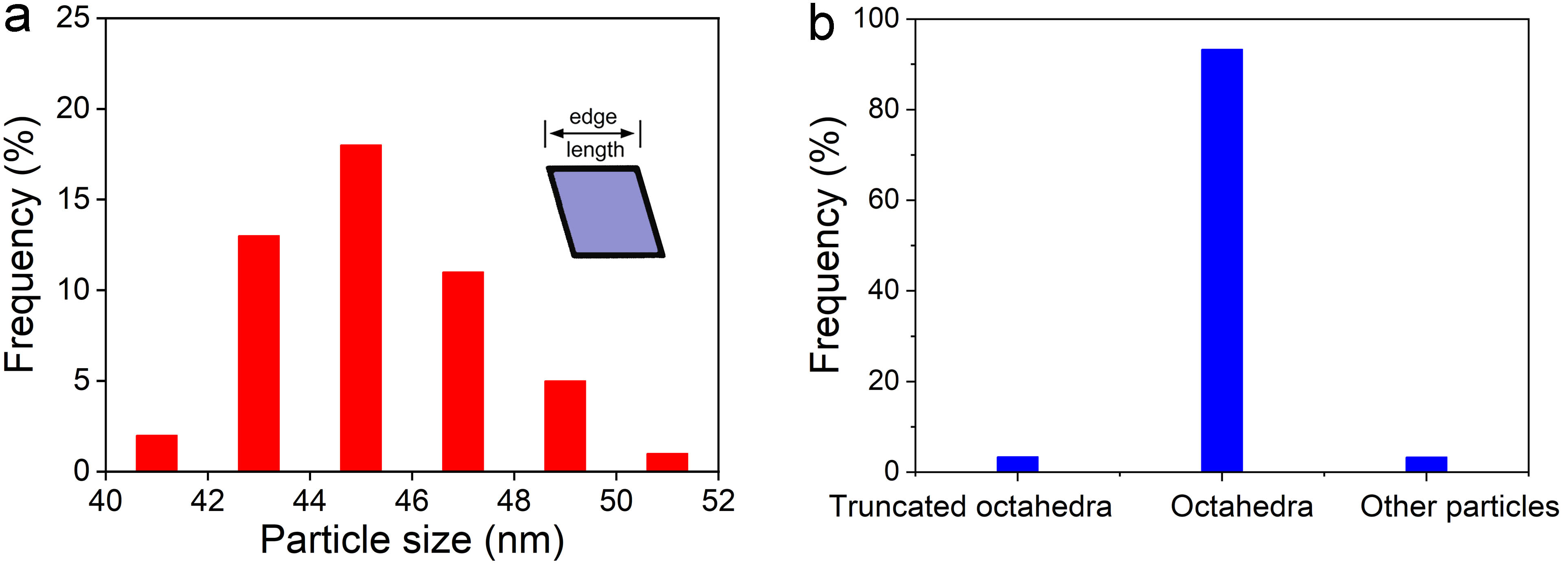


**Supplementary Figure 8 | Statistics of Fe_3_O_4_@χ-Fe_5_C_2_ octahedra from Figure 3a.** (**a**) Size distribution of Fe_3_O_4_@χ**-**Fe_5_C_2_ octahedra. The average particle size is 45.4±3.5 nm. (**b**) Contents of nanocrystals with different shapes. The purity of Fe_3_O_4_@χ**-**Fe_5_C_2_ octahedra is 93.3%.


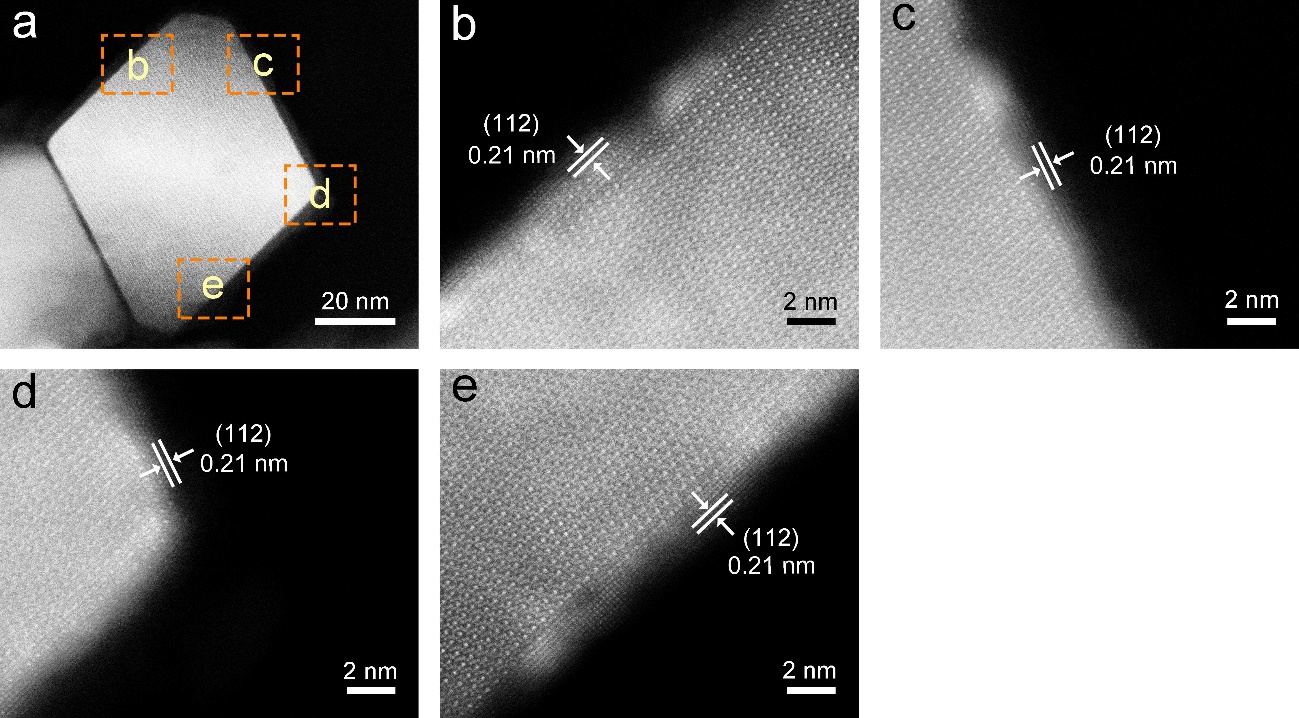


**Supplementary Figure 9 | Structural characterizations of an individual Fe_3_O_4_@χ-Fe_5_C_2_ octahedron/SiC.** (**a**) HAADF-STEM image of an individual Fe_3_O_4_@χ-Fe_5_C_2_ octahedron/SiC. (**b-e**) Magnified HAADF-STEM images of the region marked by the corresponding boxes in panel **a**.


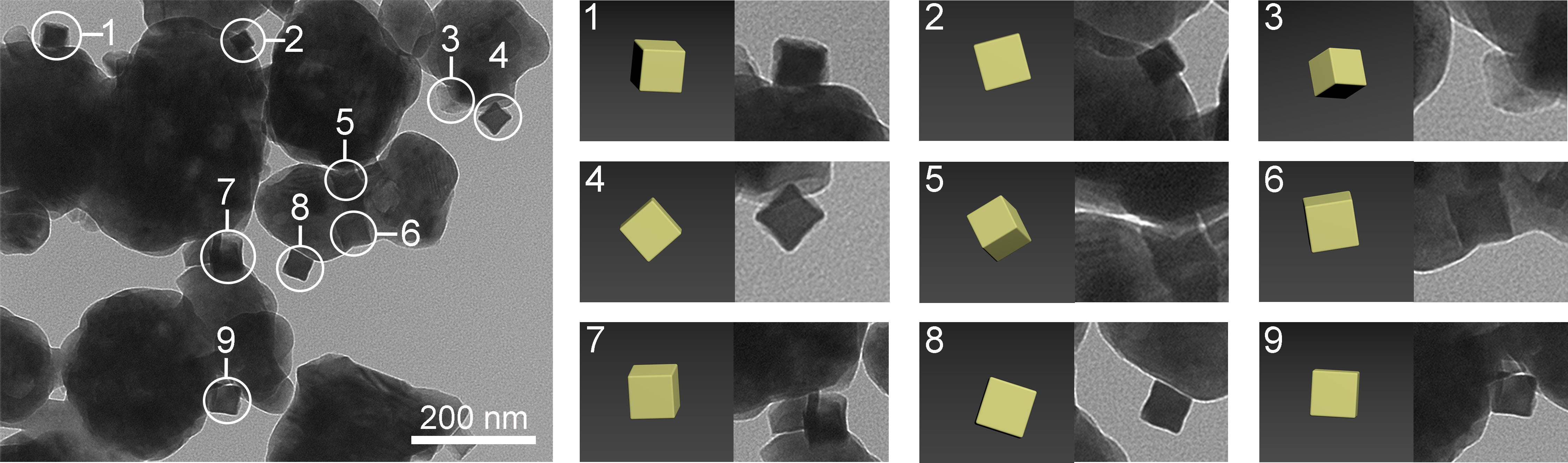


**Supplementary Figure 10 | Structural characterizations of Fe_3_O_4_@χ-Fe_5_C_2_ nanocubes/SiC.** TEM image of Fe_3_O_4_@χ-Fe_5_C_2_ nanocubes/SiC and the cubic models from different orientations.


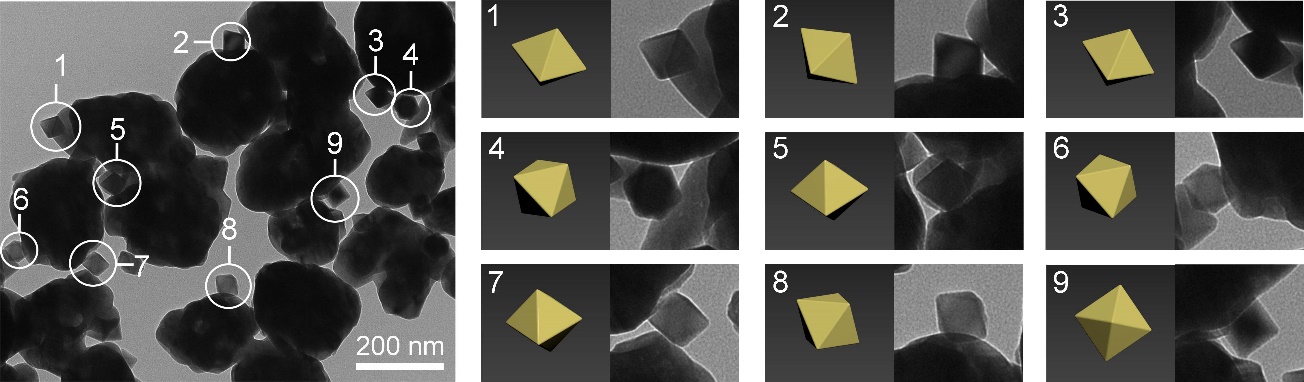


**Supplementary Figure 11 | Structural characterizations of Fe_3_O_4_@χ-Fe_5_C_2_ octahedra/SiC.** TEM image of Fe_3_O_4_@χ-Fe_5_C_2_ octahedra/SiC and the octahedral models from different orientations.

**Supplementary Table 3 | Catalytic properties of Fe_3_O_4_@χ-Fe_5_C_2_ nanocubes/SiC and octahedra/SiC towards FTS**.

| Catalysts | Conversion  (%) | CO_2_ selectivity  (%) | Product selectivity (C%, CO_2_-free) | | | | | | Carbon  balance  (%) |
| --- | --- | --- | --- | --- | --- | --- | --- | --- | --- |
|  |  |  | CH_4_ | C_2_-C_4_= | C_2_-C_4_^o^ | C_5_-C_12_= | C_5_-C_12_^o^ | C_13+_ |  |
| Fe_3_O_4_@χ-Fe_5_C_2_  nanocubes/SiC^a^ | 45.4 | 18.7 | 14.2 | 21.6 | 19.4 | 17.9 | 24.3 | 2.6 | 98.7 |
| Fe_3_O_4_@χ-Fe_5_C_2_  octahedra/SiC^a^ | 21.2 | 12.8 | 19.3 | 20.6 | 32.5 | 11.3 | 14.5 | 1.8 | 96.5 |
| χ-Fe_5_C_2_ nanoparticles/SiC^a^ | 23.6 | 10.5 | 15.5 | 29.8 | 18.4 | 16.9 | 18.0 | 1.4 | 96.8 |
| Fe_3_O_4_@χ-Fe_5_C_2_  octahedra/SiC^b^ | 42.6 | 16.3 | 20.1 | 18.3 | 22.0 | 16.0 | 21.5 | 2.1 | 97.6 |

^a^ refers to the conditions of 20 bar, syngas (64 vol% H_2_, 32 vol% CO, and 4 vol% Ar), 2400 mL h^-1^ g_cat_^-1^, and 270 ^o^C.

^b^ refers to the conditions of 20 bar, syngas (64 vol% H_2_, 32 vol% CO, and 4 vol% Ar), 800 mL h^-1^ g_cat_^-1^, and 270 ^o^C.

= refers to olefins.

^o^ refers to paraffins.


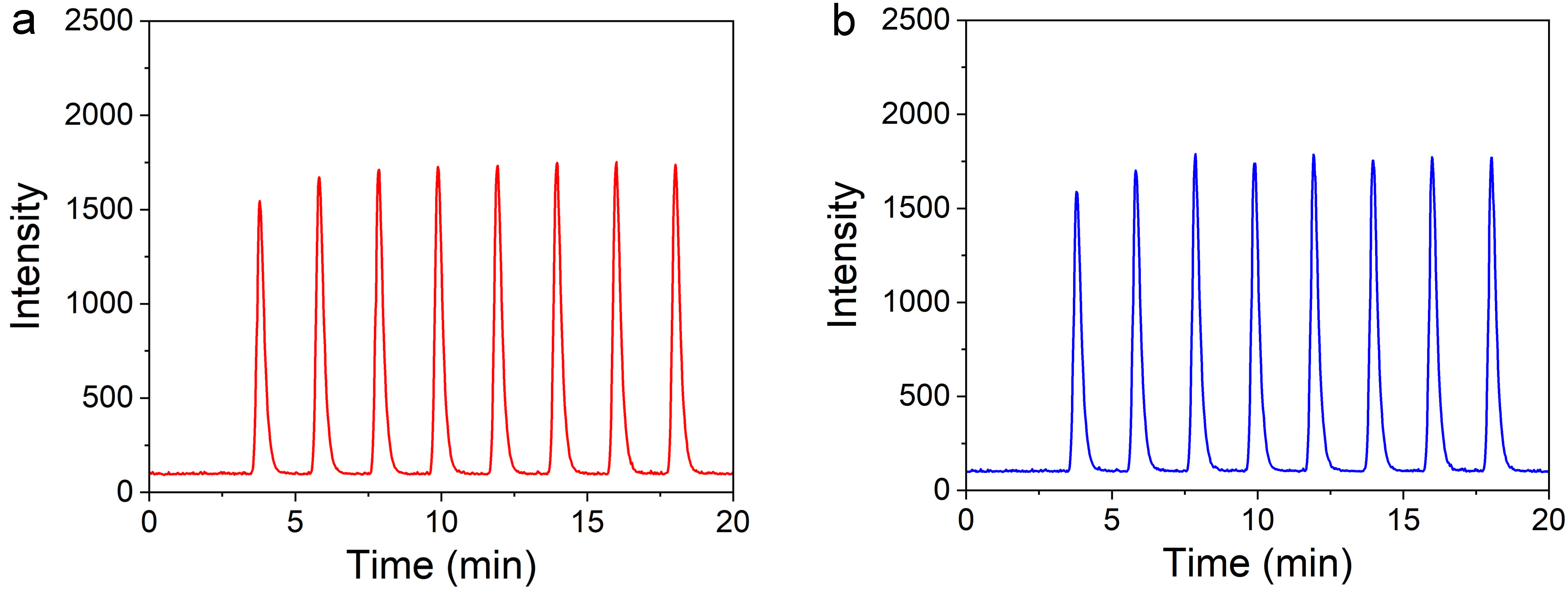


**Supplementary Figure 12 | CO pulse profiles.** CO pulse profiles of (**a**) Fe_3_O_4_@χ-Fe_5_C_2_ nanocubes/SiC and (**b**) Fe_3_O_4_@χ-Fe_5_C_2_ octahedra/SiC.


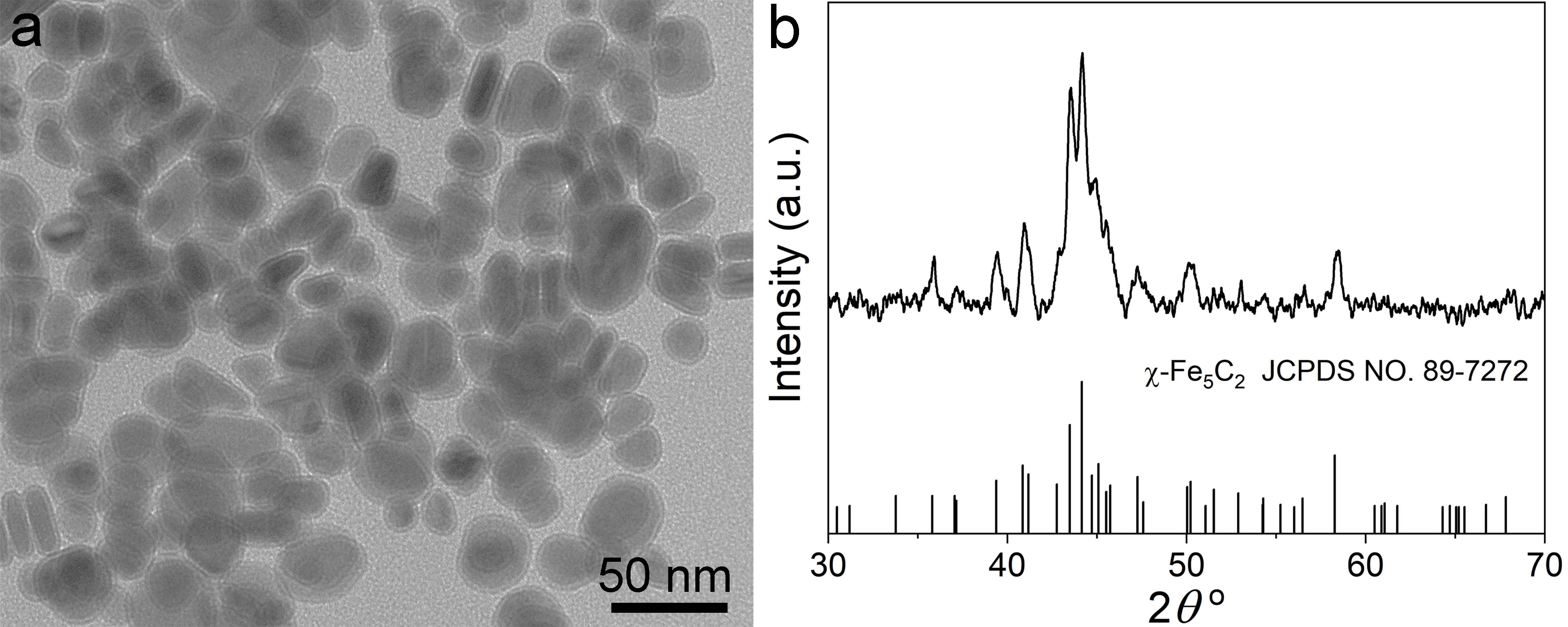


**Supplementary Figure 13 | Structural characterizations of pure χ-Fe_5_C_2_ nanoparticles.** (**a**) TEM image of pure χ-Fe_5_C_2_ nanoparticles. (**b**) XRD pattern of pure χ-Fe_5_C_2_ nanoparticles.


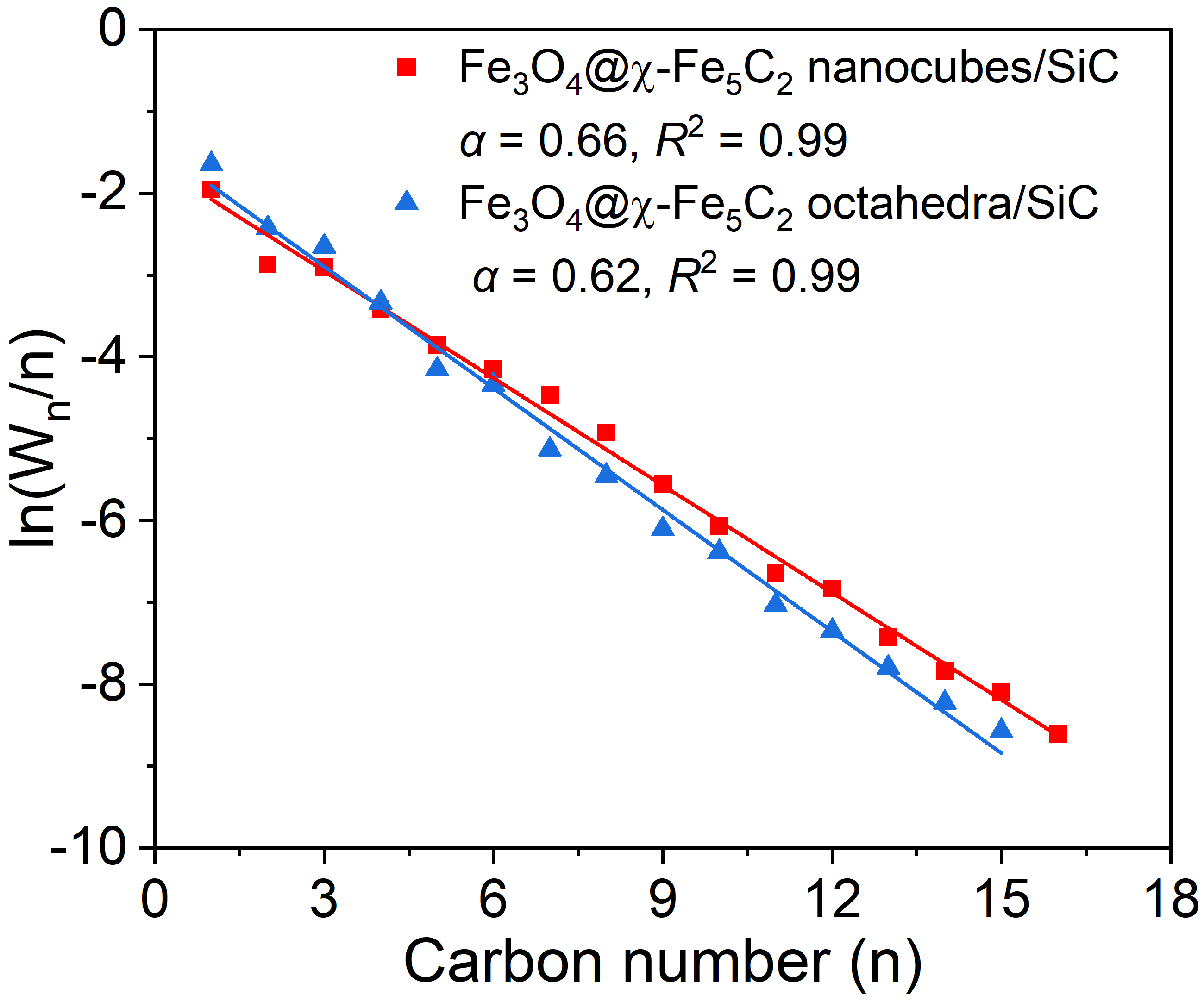


**Supplementary Figure 14 | The ASF plot and the corresponding *α* value of Fe_3_O_4_@χ-Fe_5_C_2_ nanocubes/SiC and Fe_3_O_4_@χ-Fe_5_C_2_ octahedra/SiC.** The reaction was conducted under 20 bar of syngas (CO:H_2_ = 1:2, 2400 mL h^-1^ g_cat_^-1^) at 270 ^o^C.


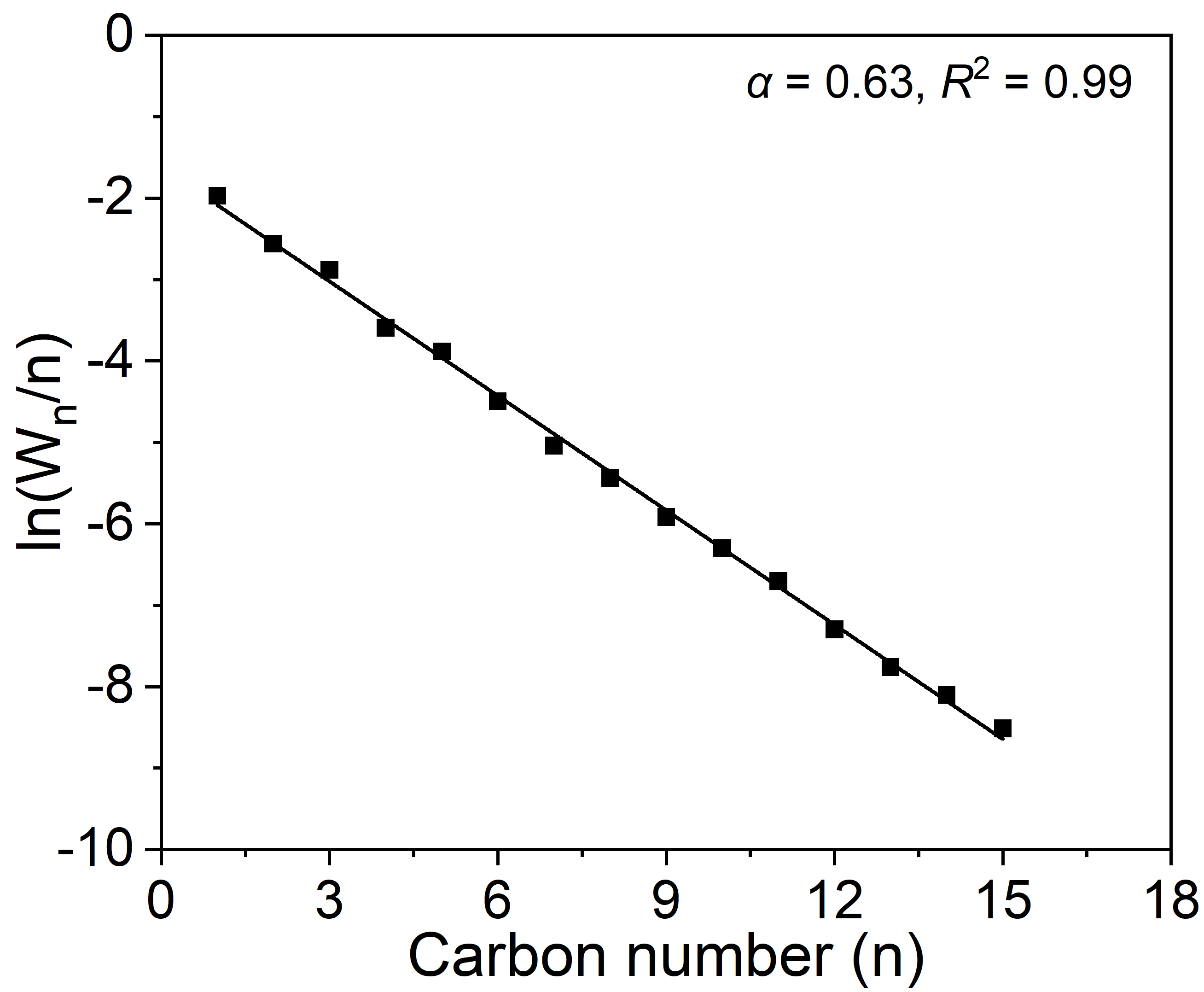


**Supplementary Figure 15 | The ASF plot and the corresponding *α* value of χ-Fe_5_C_2_ nanoparticles/SiC.** The reaction was conducted under 20 bar of syngas (CO:H_2_ = 1:2, 2400 mL h^-1^ g_cat_^-1^) at 270 ^o^C.


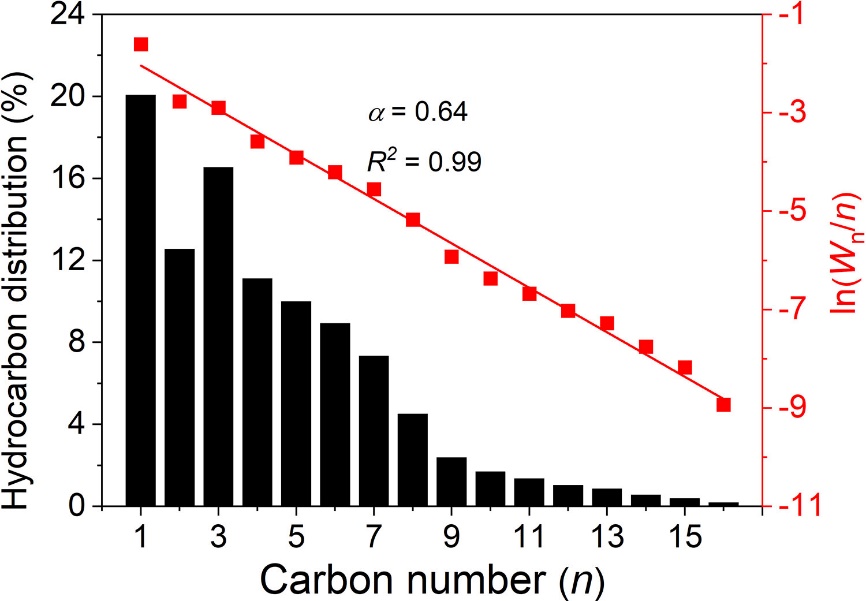


**Supplementary Figure 16 | Hydrocarbon distribution, the ASF plot, and the corresponding *α* value of Fe_3_O_4_@χ-Fe_5_C_2_ octahedra/SiC at similar conversion levels of Fe_3_O_4_@χ-Fe_5_C_2_ nanocubes/SiC.** The reaction was conducted under 20 bar of syngas (CO:H_2_ = 1:2, 800 mL h^-1^ g_cat_^-1^) at 270 ^o^C.


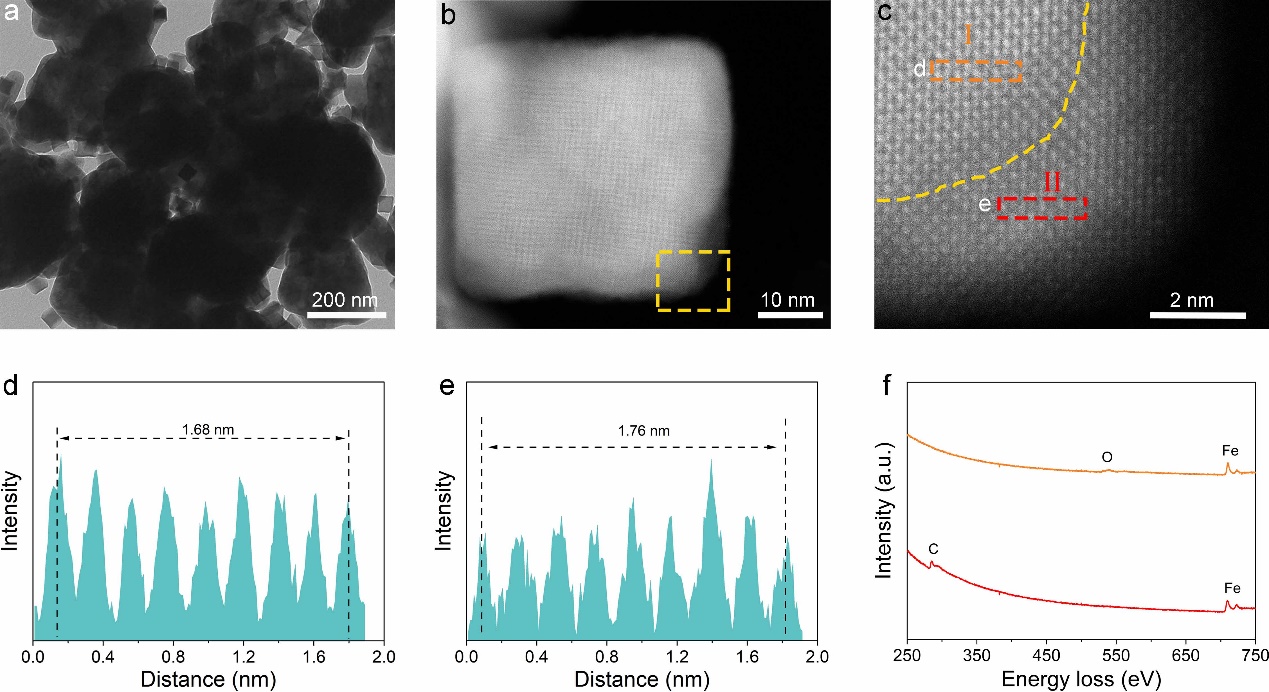


**Supplementary Figure 17 | Structural characterizations of Fe_3_O_4_@χ-Fe_5_C_2_ nanocubes after 100 h on stream**. (**a**) TEM image of Fe_3_O_4_@χ-Fe_5_C_2_ nanocube/SiC. (**b**) HAADF-STEM image of an individual Fe_3_O_4_@χ-Fe_5_C_2_ nanocube. (**c**) Magnified HAADF-STEM image of the region marked by the corresponding boxes in panel **b**. (**d**) Intensity profile recorded from the area indicated by the rectangular box in panel **c**. (**e**) Intensity profile recorded from the area indicated by the rectangular box in panel **c**. (**f**) EELS spectra of a Fe_3_O_4_@χ-Fe_5_C_2_ nanocube in panel **c**.


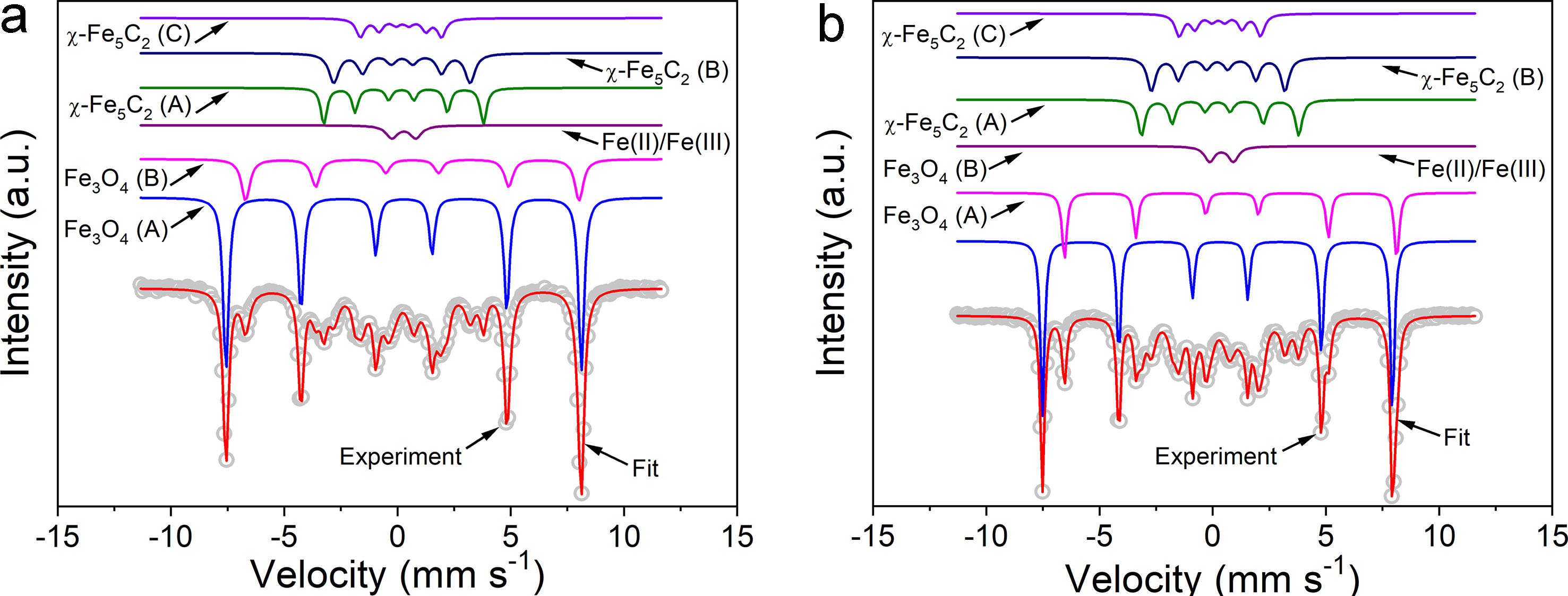


**Supplementary Figure 18 | Mössbauer spectra characterizations.** (**a**) Mössbauer spectra of Fe_3_O_4_@χ-Fe_5_C_2_ nanocubes after 100 h on stream. (**b**) Mössbauer spectra of Fe_3_O_4_@χ-Fe_5_C_2_ octahedra after 100 h on stream.

**Supplementary Table 4 | Mössbauer parameters of Fe_3_O_4_@χ-Fe_5_C_2_ nanocubes and Fe_3_O_4_@χ-Fe_5_C_2_ octahedra after 100 h on stream**. The isomer shift (IS), quadrupole splitting (QS), hyperfine field, and spectral contribution are given.

| Samples | Phase ascription | Mössbaure parameters | | | |
| --- | --- | --- | --- | --- | --- |
|  |  | IS (mm/s) | QS (mm/s) | Hyperfine field (T) | Spectral contribution |
| Fe_3_O_4_@χ-Fe_5_C_2_ nanocubes after reaction | Fe_3_O_4_ (A) | 0.30 | -0.01 | 48.9 | 40.7% |
|  | Fe_3_O_4_ (B) | 0.65 | 0.01 | 45.5 | 15.4% |
|  | χ-Fe_5_C_2_ (A) | 0.22 | 0.11 | 21.8 | 12.9% |
|  | χ-Fe_5_C_2_ (B) | 0.17 | 0.06 | 18.6 | 17.0% |
|  | χ-Fe_5_C_2_ (C) | 0.20 | 0.02 | 11.1 | 9.9% |
|  | Fe(II)/Fe(III) | 0.34 | 1.07 | - | 4.1% |
| Fe_3_O_4_@χ-Fe_5_C_2_ octahedra after reaction | Fe_3_O_4_ (A) | 0.33 | -0.05 | 48.5 | 38.7% |
|  | Fe_3_O_4_ (B) | 0.78 | -0.09 | 45.4 | 16.6% |
|  | χ-Fe_5_C_2_ (A) | 0.27 | 0.11 | 21.6 | 14.5% |
|  | χ-Fe_5_C_2_ (B) | 0.22 | 0.04 | 18.3 | 15.5% |
|  | χ-Fe_5_C_2_ (C) | 0.28 | 0.06 | 11.2 | 10.4% |
|  | Fe(II)/Fe(III) | 0.39 | 1.04 | - | 4.3% |


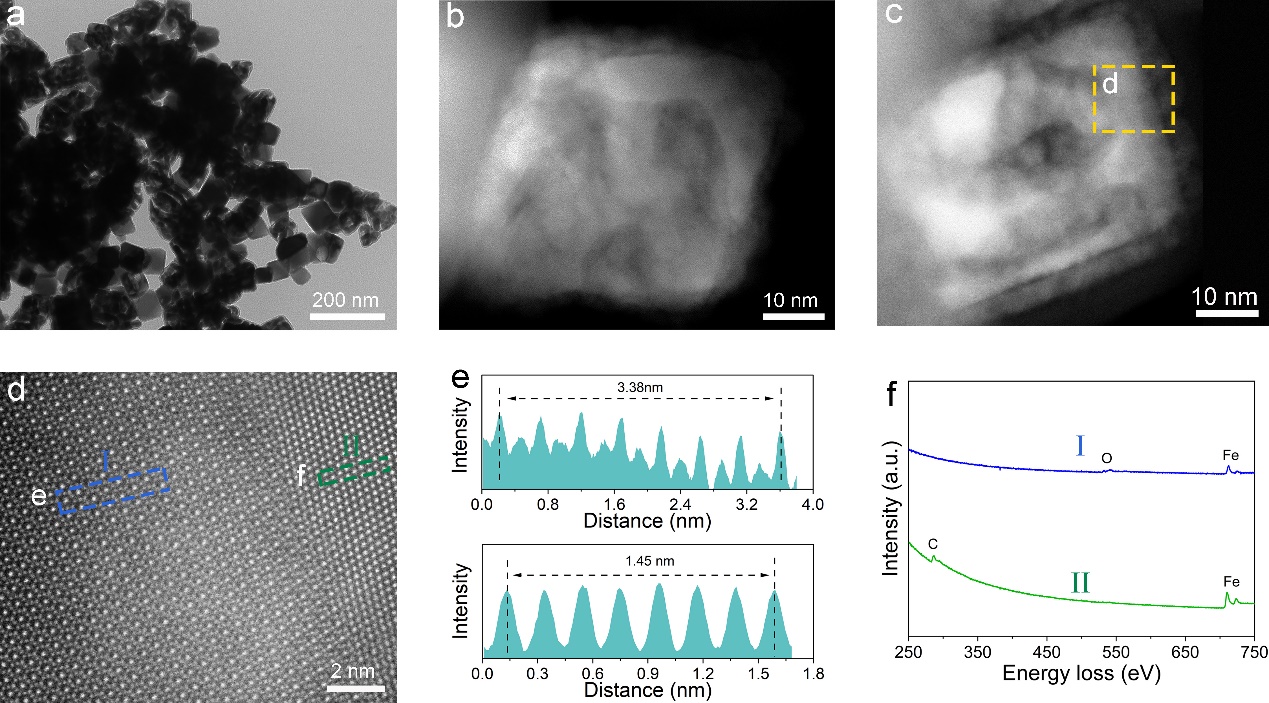


**Supplementary Figure 19 | Structural characterizations of Fe_3_O_4_@χ-Fe_5_C_2_ octahedra after 100 h on stream.** (**a**) TEM image of Fe_3_O_4_@χ-Fe_5_C_2_ octahedra/SiC. (**b**) HAADF-STEM image of an individual Fe_3_O_4_@χ-Fe_5_C_2_ octahedron. (**c**) HAADF-STEM image of another Fe_3_O_4_@χ-Fe_5_C_2_ octahedron. (**c**) Magnified HAADF-STEM image of the region marked by the corresponding boxes in panel **b**. (**d**) Intensity profile recorded from the area indicated by the rectangular box in panel **d**. (**e**) Intensity profile recorded from the area indicated by the rectangular box in panel **d**. (**f**) EELS spectra of a Fe_3_O_4_@χ-Fe_5_C_2_ octahedron in panel **d**.


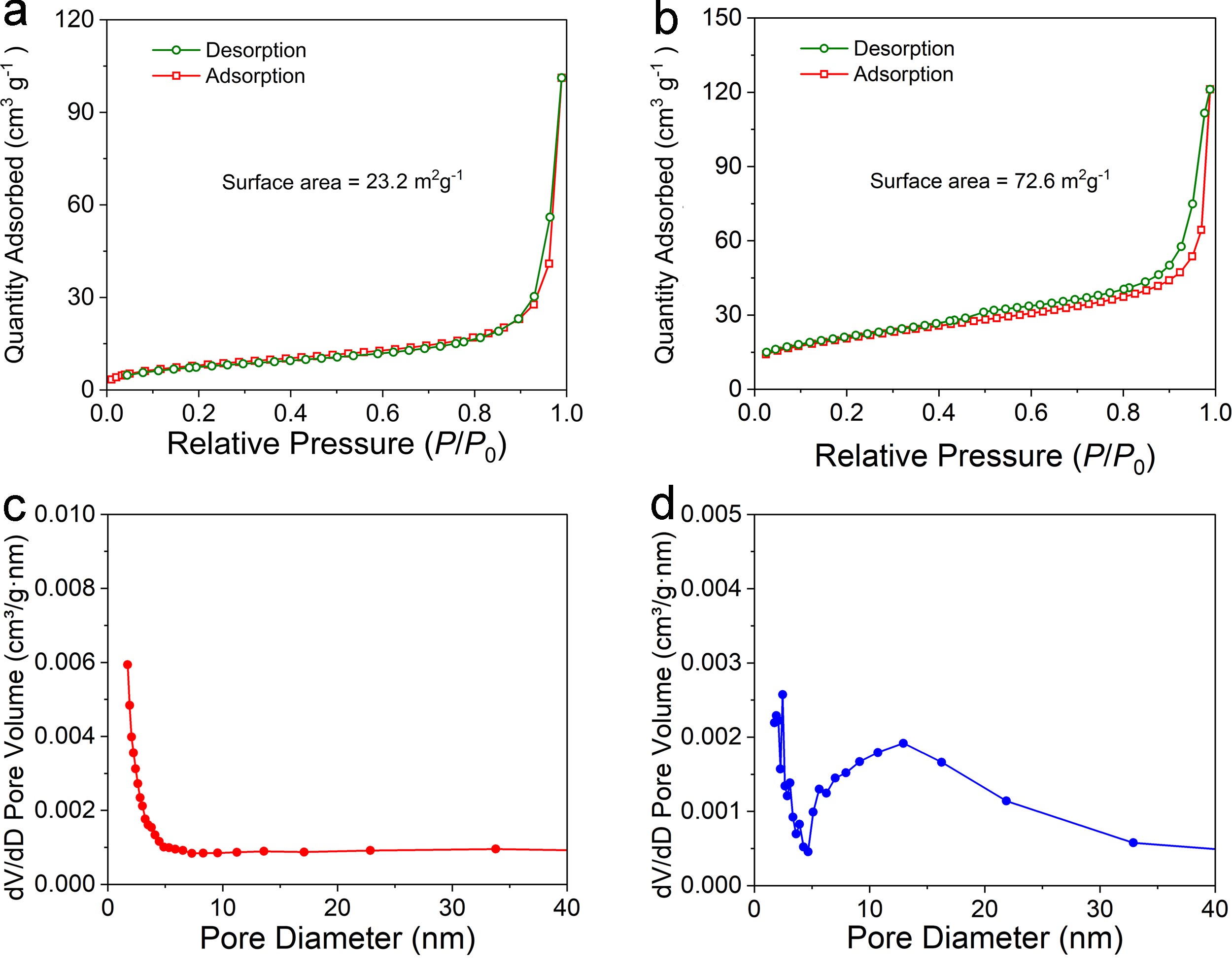


**Supplementary Figure 20 | Textural properties of Fe_3_O_4_@χ-Fe_5_C_2_ nanocubes and octahedra after 100 h on stream. (a, b)** Nitrogen adsorption and desorption isotherm of (**a**) Fe_3_O_4_@χ-Fe_5_C_2_ nanocubes and (**b**) Fe_3_O_4_@χ-Fe_5_C_2_ octahedra after 100 h on stream. (**c, d**) Pore-size distributions of (**c**) Fe_3_O_4_@χ-Fe_5_C_2_ nanocubes and (**d**) Fe_3_O_4_@χ-Fe_5_C_2_ octahedra after 100 h on stream derived from the nitrogen adsorption-desorption isotherms by the BJH method.


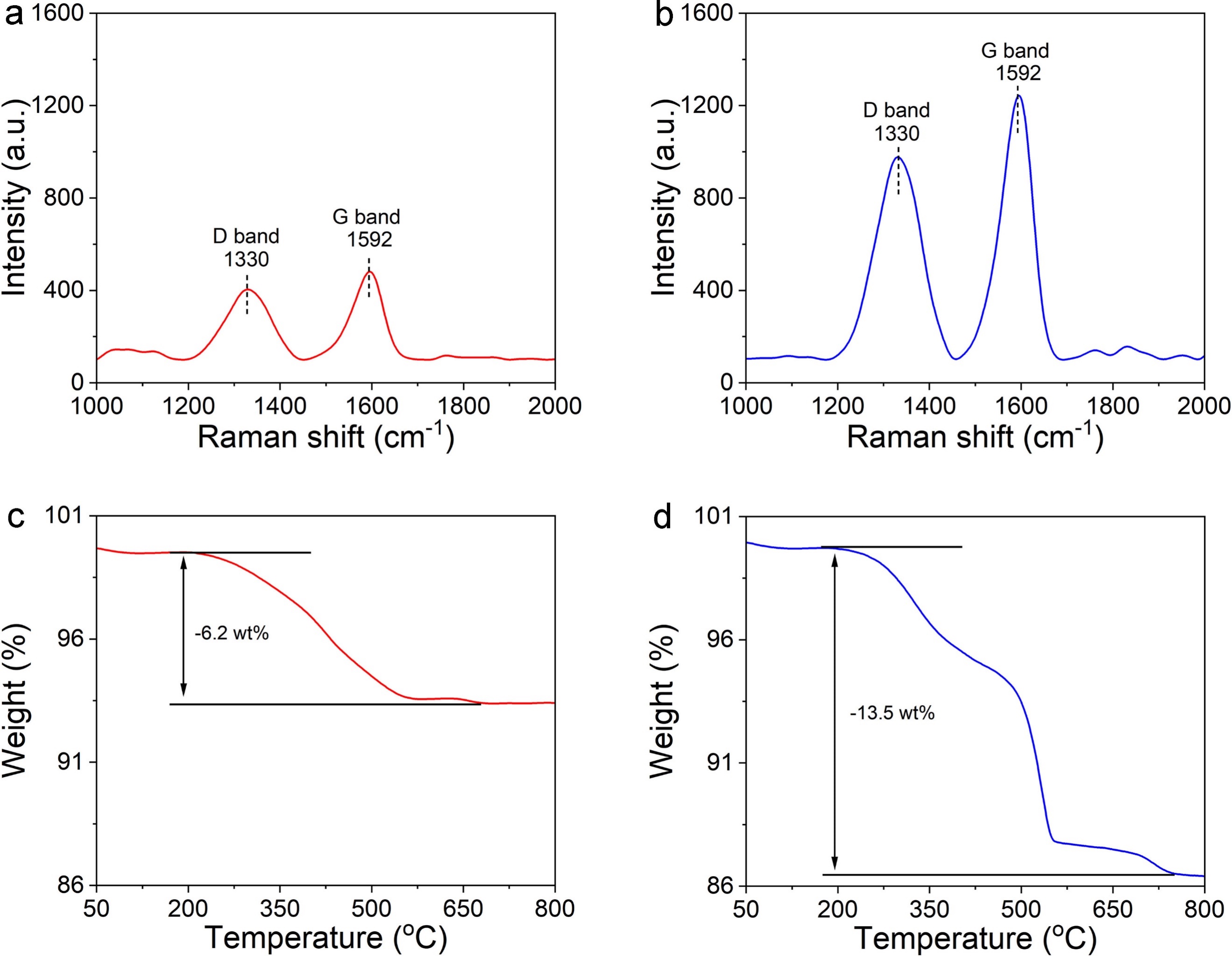


**Supplementary Figure 21 |** Raman spectra of Fe_3_O_4_@χ-Fe_5_C_2_ nanocubes (**a**) and Fe_3_O_4_@χ-Fe_5_C_2_ octahedra (**b**) after 100 h on stream. TGA profiles of Fe_3_O_4_@χ-Fe_5_C_2_ nanocubes (**c**) and Fe_3_O_4_@χ-Fe_5_C_2_ octahedra (**d**) after 100 h on stream.


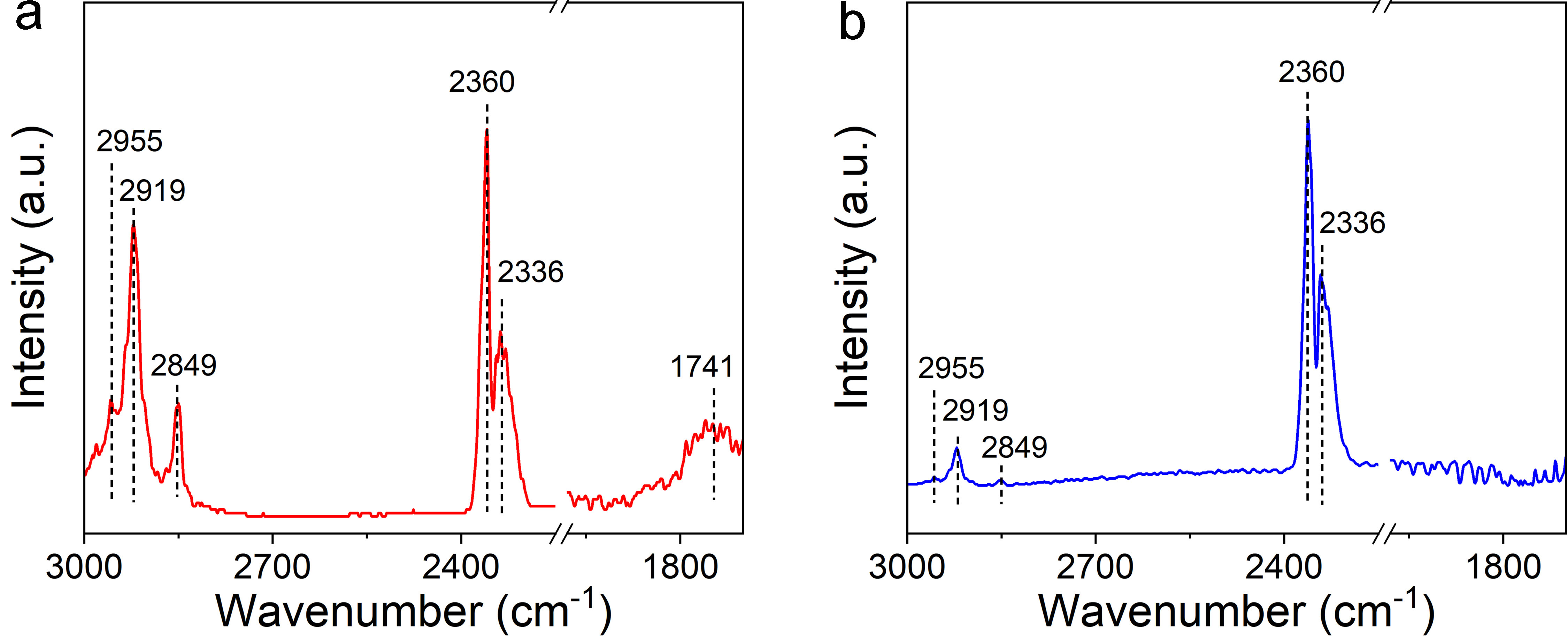


**Supplementary Figure 22 |** (**a**) *In-situ* DRIFTS spectra of Fe_3_O_4_@χ-Fe_5_C_2_ nanocubes and (**b**) Fe_3_O_4_@χ-Fe_5_C_2_ octahedra after being exposed to 20 bar of syngas for 30 min at 270 ^o^C.

**Supplementary Table 5 |Assignment of DRIFTS peaks.**

| Wavenumber (cm^-1^) | Assignment | References |
| --- | --- | --- |
| 2360, 2336 | Gaseous CO_2_ | 1 |
| 2955 | Asymmetrical stretching vibration of C-H bonds in CH_3_* | 2, 3 |
| 2919 | Asymmetrical stretching vibration of C-H bonds in CH_2_* | 4 |
| 2849 | Symmetrical stretching vibration of C-H bonds in CH_2_* | 5 |
| 1741 | Stretching vibration of C=O bonds in CHO* | 6,7 |


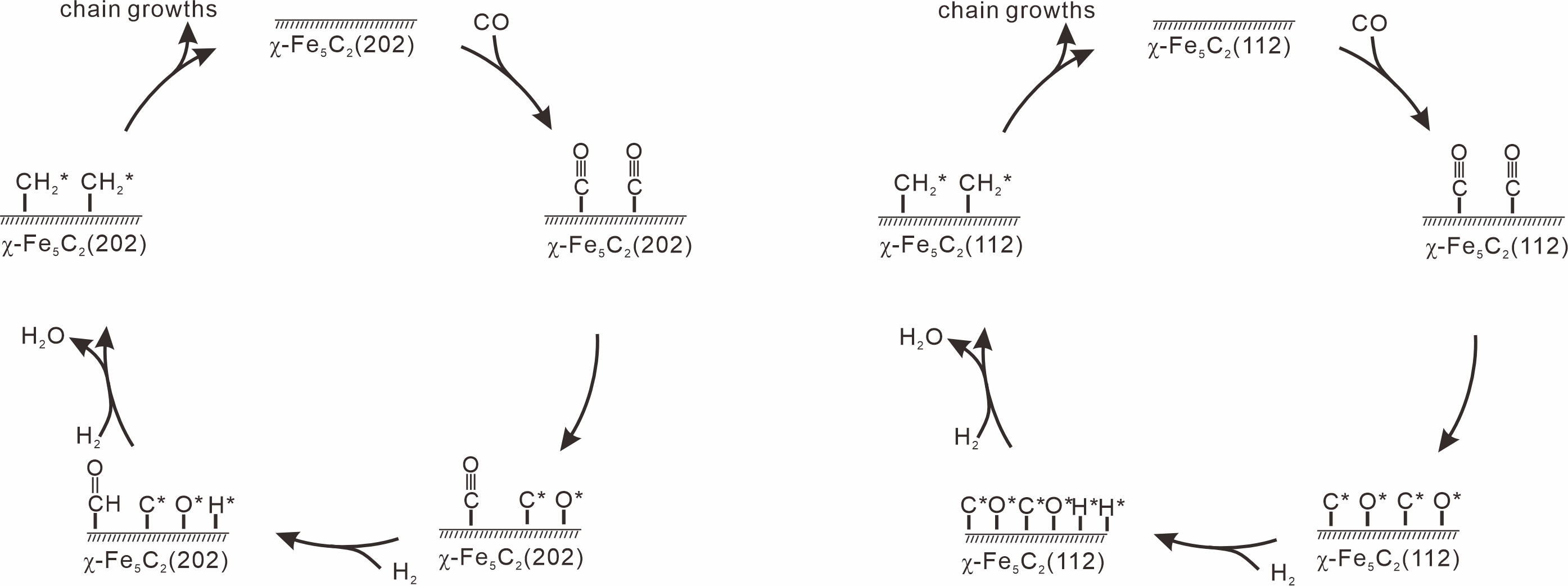


**Supplementary Figure 23 |** The schematic diagram for different CO dissociation pathways of Fe_3_O_4_@χ-Fe_5_C_2_ nanocubes and Fe_3_O_4_@χ-Fe_5_C_2_ octahedra.


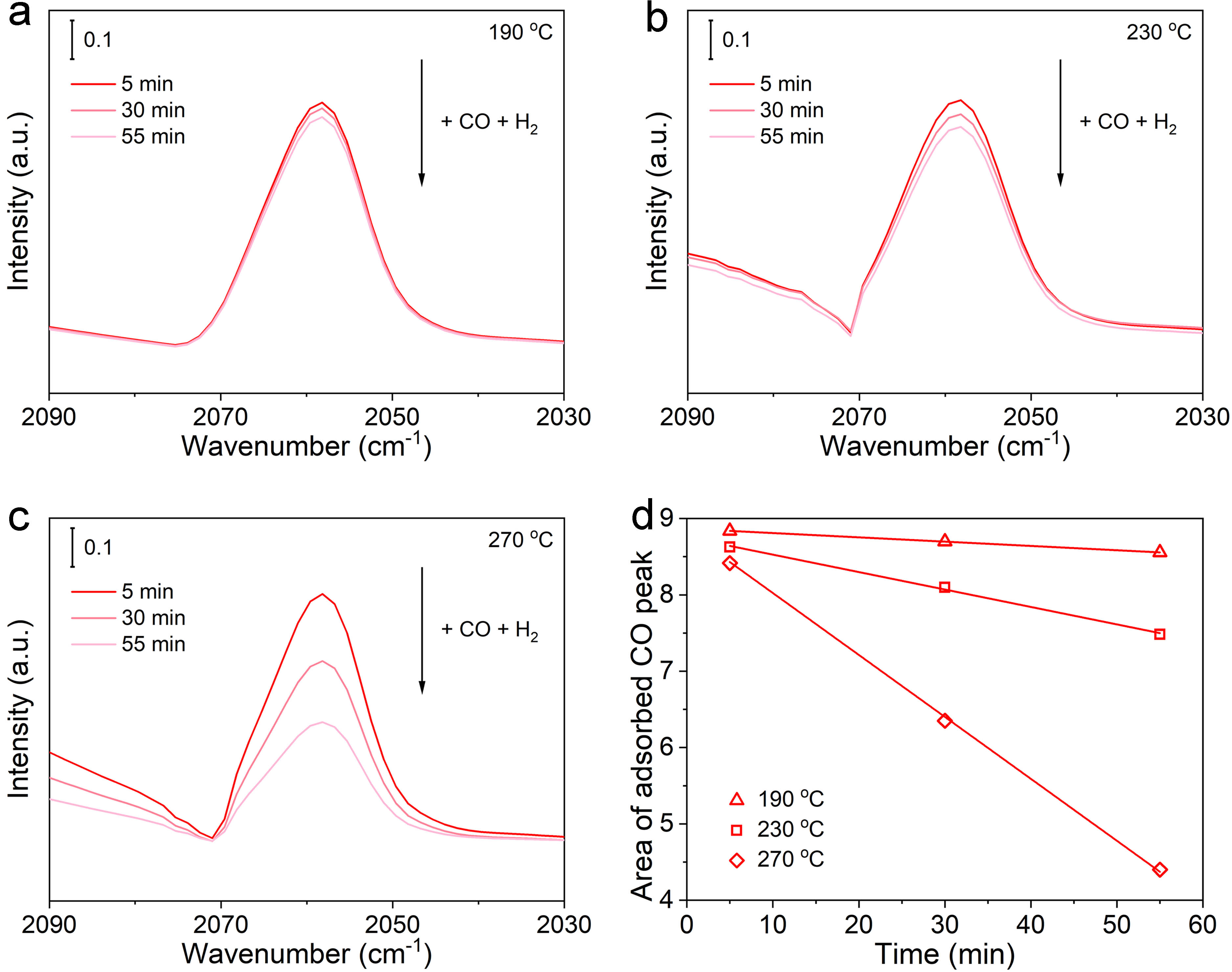


**Supplementary Figure 24 | Mechanistic studies of Fe_3_O_4_@χ-Fe_5_C_2_ nanocubes for CO dissociation**. (**a-c**) *In-situ* DRIFTS spectra of CO adsorption on Fe_3_O_4_@χ-Fe_5_C_2_ nanocubes after being exposed to CO and purged with H_2_ at (**a**) 190 ^o^C, (**b**) 230 ^o^C, and (**c**) 270 ^o^C. Specifically, Fe_3_O_4_@χ-Fe_5_C_2_ nanocubes were exposed to 1 bar of CO for 30 min. Subsequently, the spectra were recorded at 5, 30, and 55 min after being purged H_2_. (**d**) Time course of the area of adsorbed CO peak over Fe_3_O_4_@χ-Fe_5_C_2_ nanocubes in H_2_.


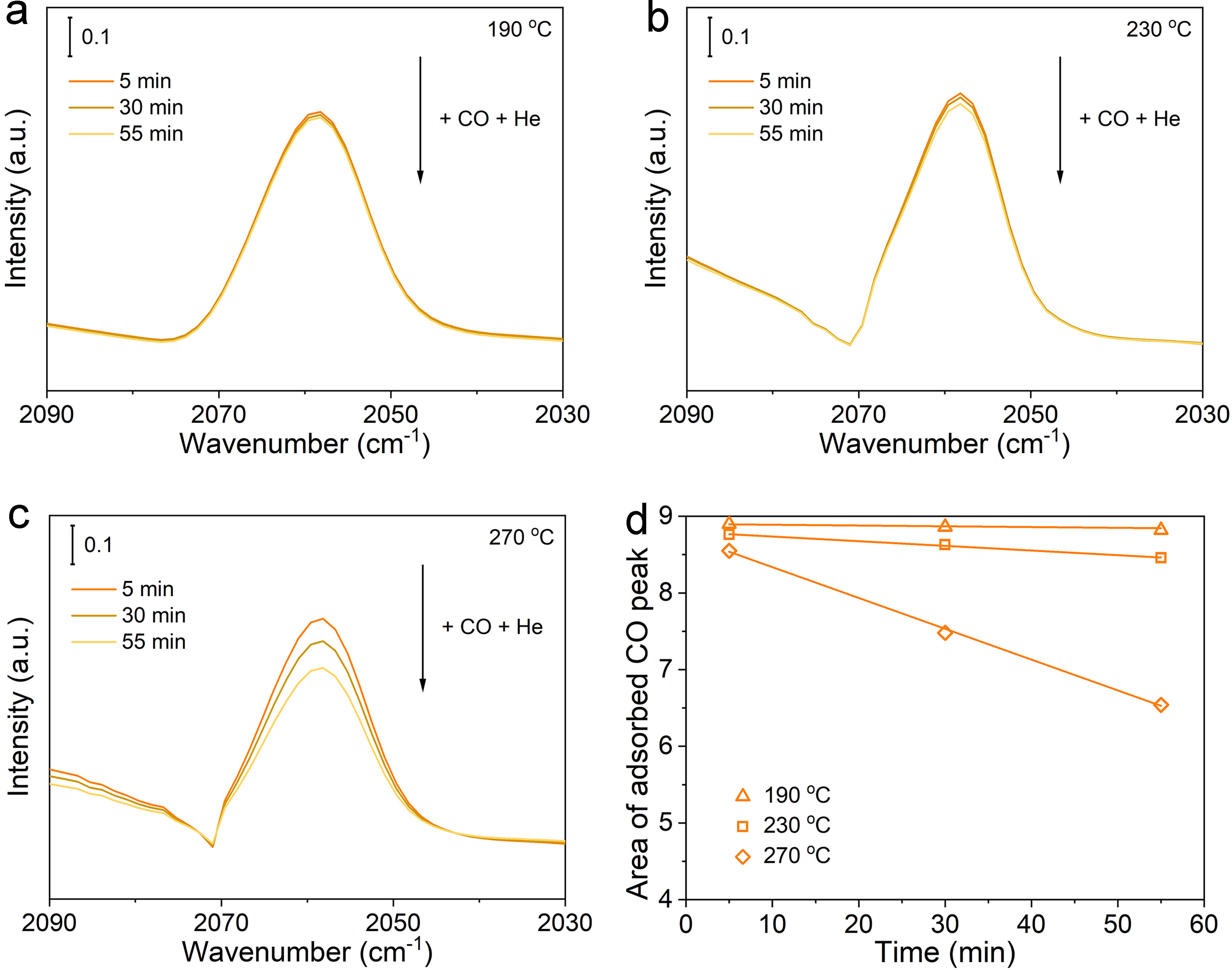


**Supplementary Figure 25 | Mechanistic studies of Fe_3_O_4_@χ-Fe_5_C_2_ nanocubes for CO dissociation**. (**a-c**) *In-situ* DRIFTS spectra of CO adsorption on Fe_3_O_4_@χ-Fe_5_C_2_ nanocubes after being exposed to CO and purged with He at (**a**) 190 ^o^C, (**b**) 230 ^o^C, and (**c**) 270 ^o^C. Specifically, Fe_3_O_4_@χ-Fe_5_C_2_ nanocubes were exposed to 1 bar of CO for 30 min. Subsequently, the spectra were recorded at 5, 30, and 55 min after being purged He. (**d**) Time course of the area of adsorbed CO peak over Fe_3_O_4_@χ-Fe_5_C_2_ nanocubes in He.


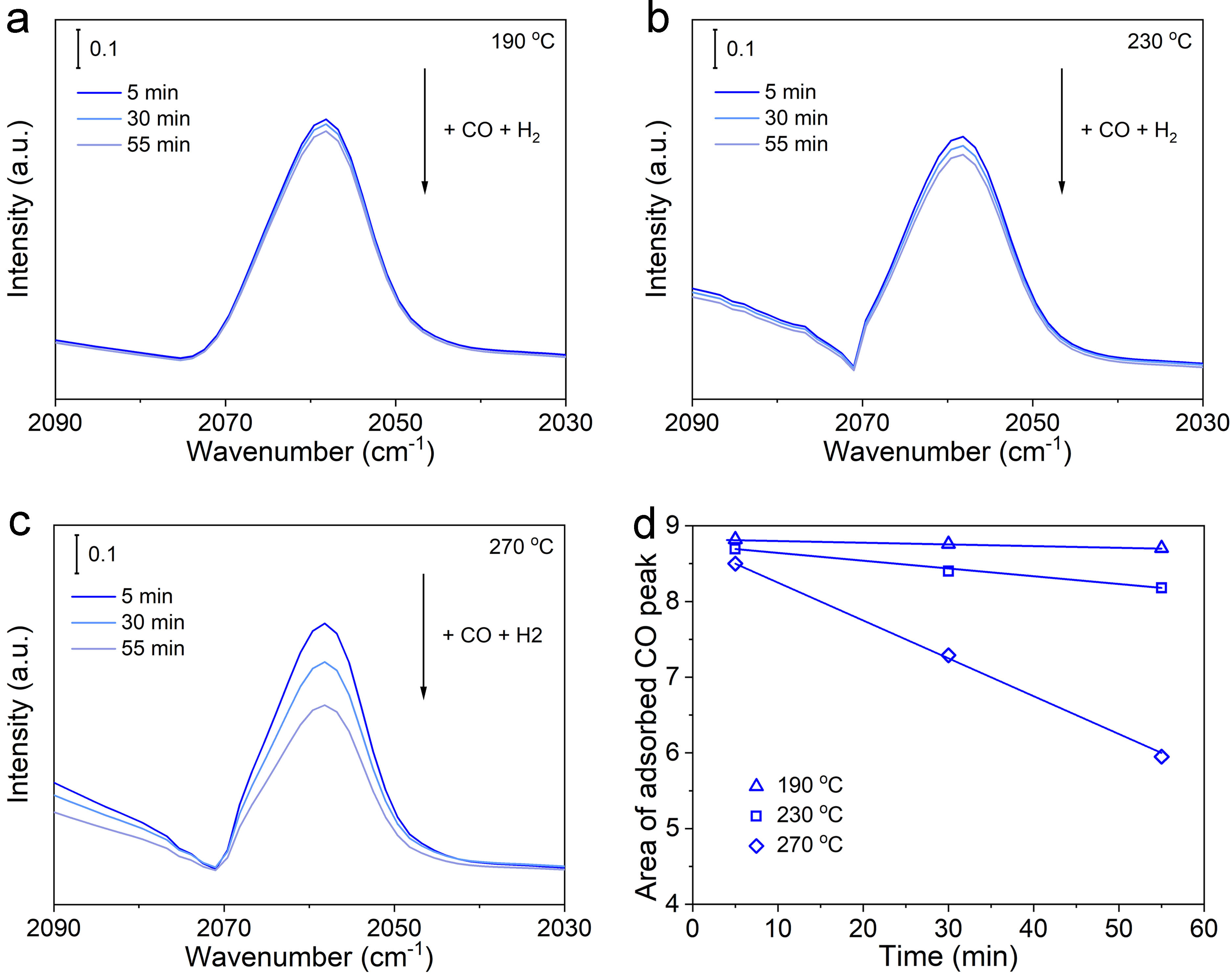


**Supplementary Figure 26 | Mechanistic studies of Fe_3_O_4_@χ-Fe_5_C_2_ octahedra for CO dissociation**. (**a-c**) *In-situ* DRIFTS spectra of CO adsorption on Fe_3_O_4_@χ-Fe_5_C_2_ octahedra after being exposed to CO and purged with H_2_ at (**a**) 190 ^o^C, (**b**) 230 ^o^C, and (**c**) 270 ^o^C. Specifically, Fe_3_O_4_@χ-Fe_5_C_2_ octahedra were exposed to 1 bar of CO for 30 min. Subsequently, the spectra were recorded at 5, 30, and 55 min after being purged H_2_. (**d**) Time course of the area of adsorbed CO peak over Fe_3_O_4_@χ-Fe_5_C_2_ octahedra in H_2_.


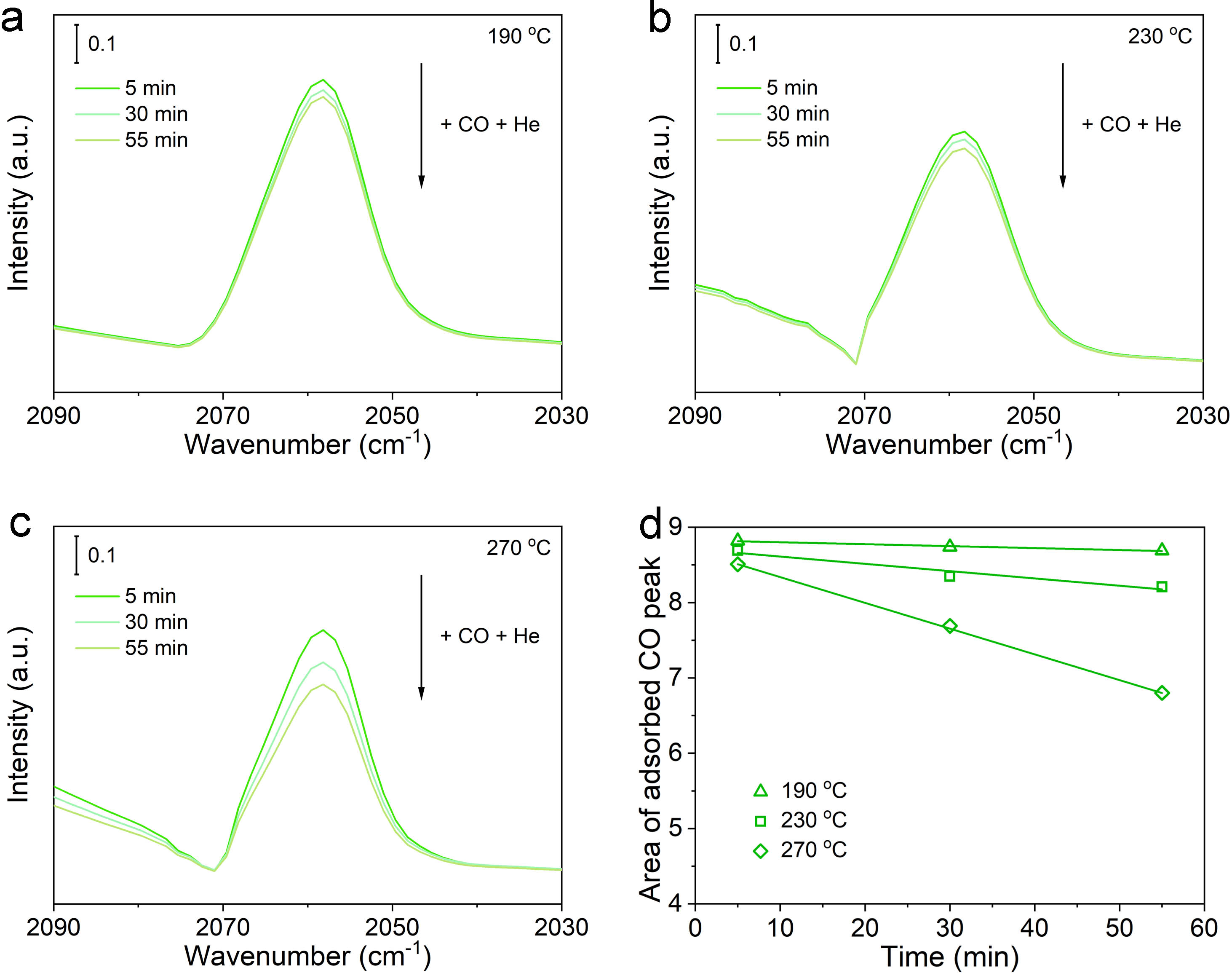


**Supplementary Figure 27 | Mechanistic studies of Fe_3_O_4_@χ-Fe_5_C_2_ octahedra for CO dissociation**. (**a-c**) *In-situ* DRIFTS spectra of CO adsorption on Fe_3_O_4_@χ-Fe_5_C_2_ octahedra after being exposed to CO and purged with He at (**a**) 190 ^o^C, (**b**) 230 ^o^C, and (**c**) 270 ^o^C. Specifically, Fe_3_O_4_@χ-Fe_5_C_2_ octahedra were exposed to 1 bar of CO for 30 min. Subsequently, the spectra were recorded at 5, 30, and 55 min after being purged He. (**d**) Time course of the area of adsorbed CO peak over Fe_3_O_4_@χ-Fe_5_C_2_ octahedra in He.


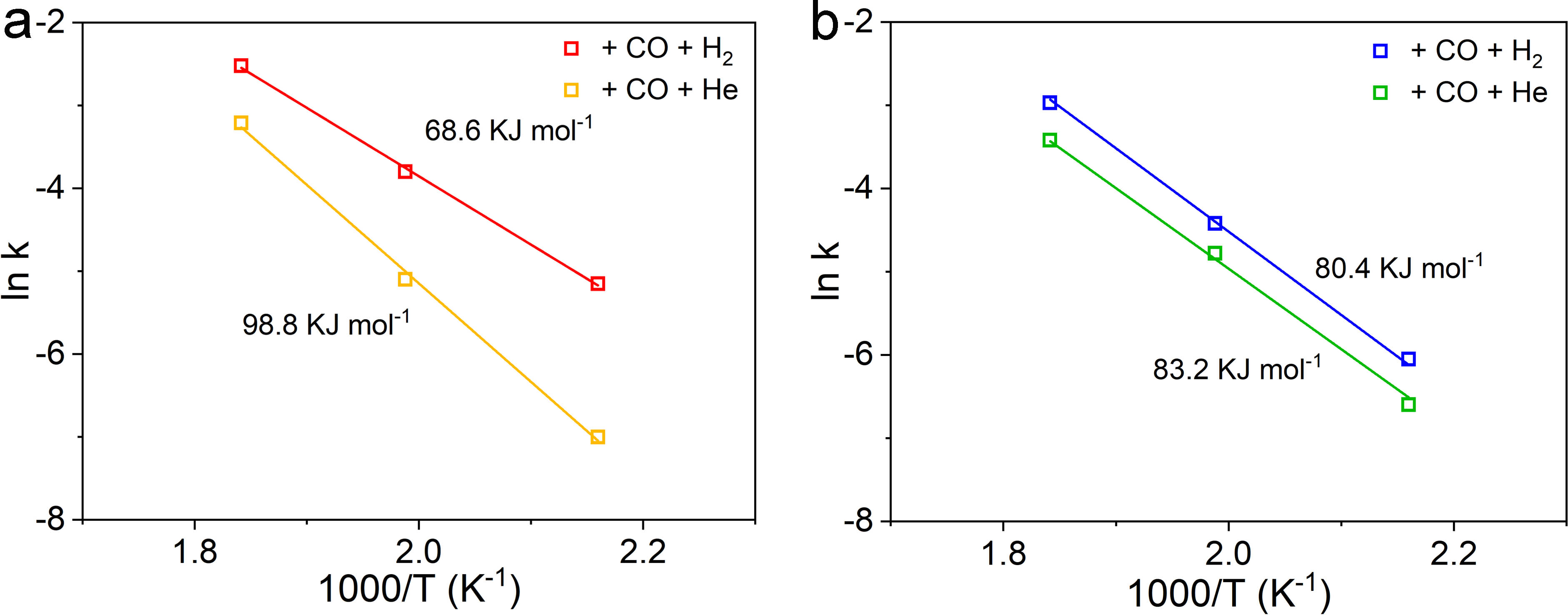


**Supplementary Figure 28 |** (**a, b**) Arrhenius plots and activation energy of (**a**) Fe_3_O_4_@χ-Fe_5_C_2_ nanocubes and (**b**) Fe_3_O_4_@χ-Fe_5_C_2_ octahedra after being exposed to CO and purged with He or H_2_.


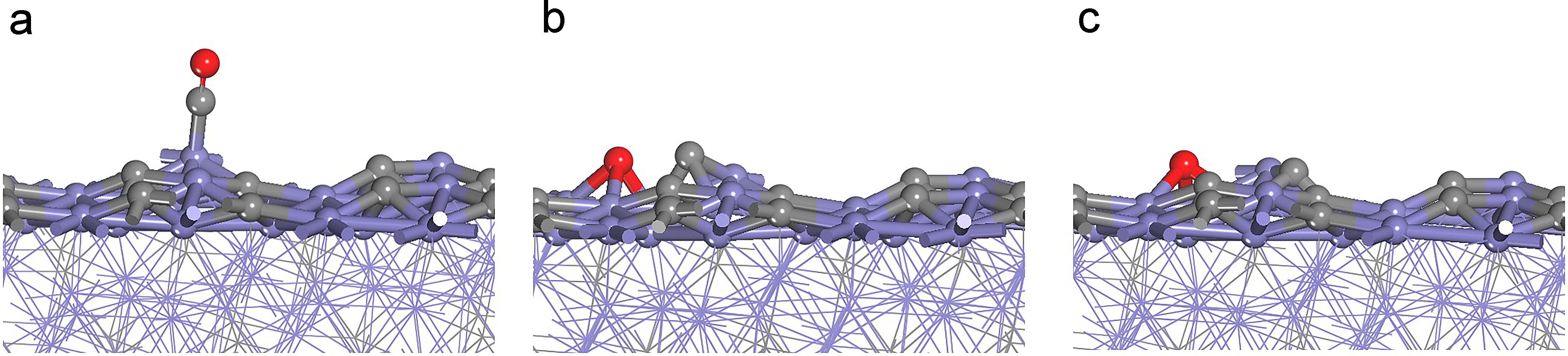


**Supplementary Figure 29 | Calculated structure models of the direct CO dissociation route on the χ-Fe_5_C_2_(202) facet.** Models of (**a**) CO*, (**b**) TS, and (**c**) C* + O*.


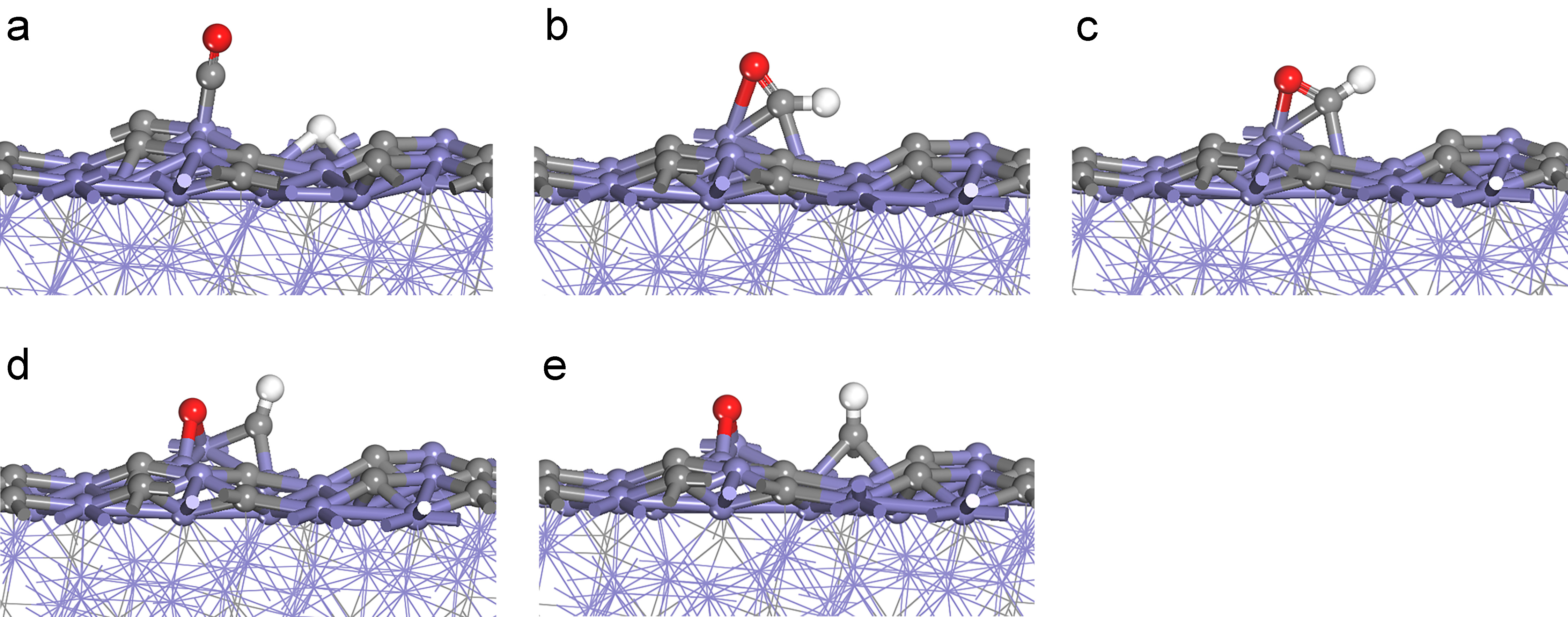


**Supplementary Figure 30 |** **Calculated structure models of the hydrogen-assisted CO dissociation route on the χ-Fe_5_C_2_(202) facet.** Models of (**a**) CO* + H*, (**b**) TS1, (**c**) HCO*, (**d**) TS2, and (**e**) HC* + O*.

**
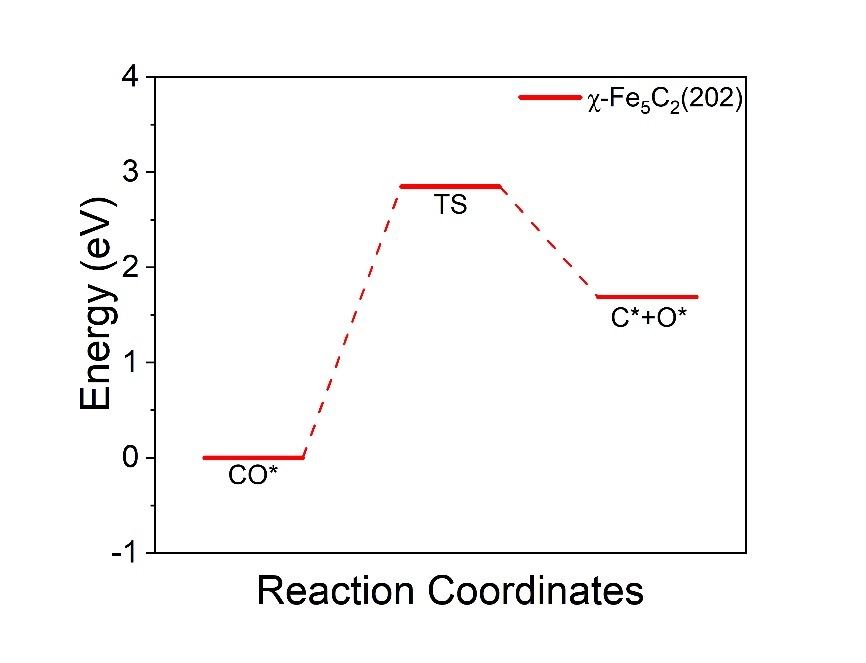
**

**Supplementary Figure 31 |** **Energy barrier of the direct CO dissociation route on the χ-Fe_5_C_2_(202) facet.**

**
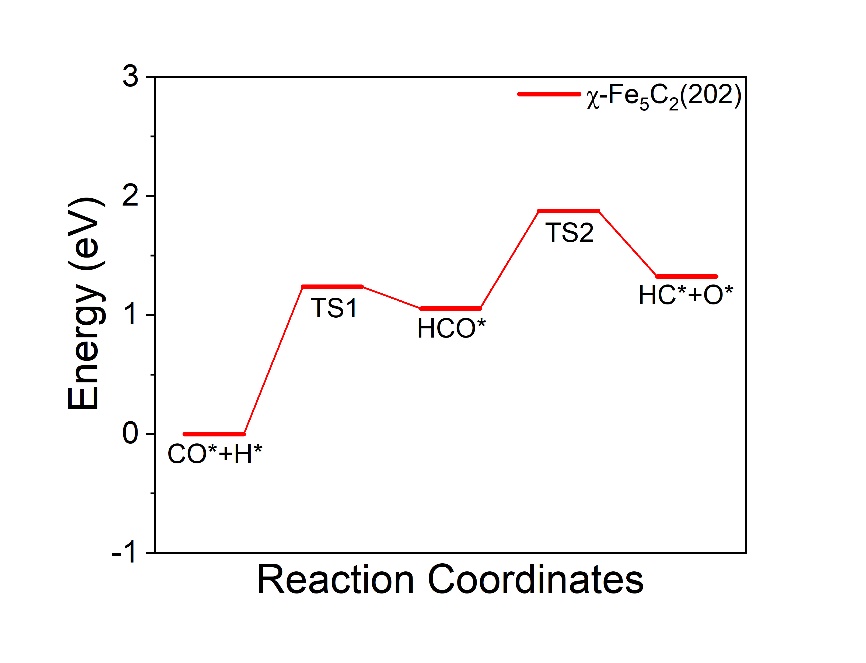
**

**Supplementary Figure 32 |** **Energy barrier of the hydrogen-assisted CO dissociation route on the χ-Fe_5_C_2_(202) facet.**

**
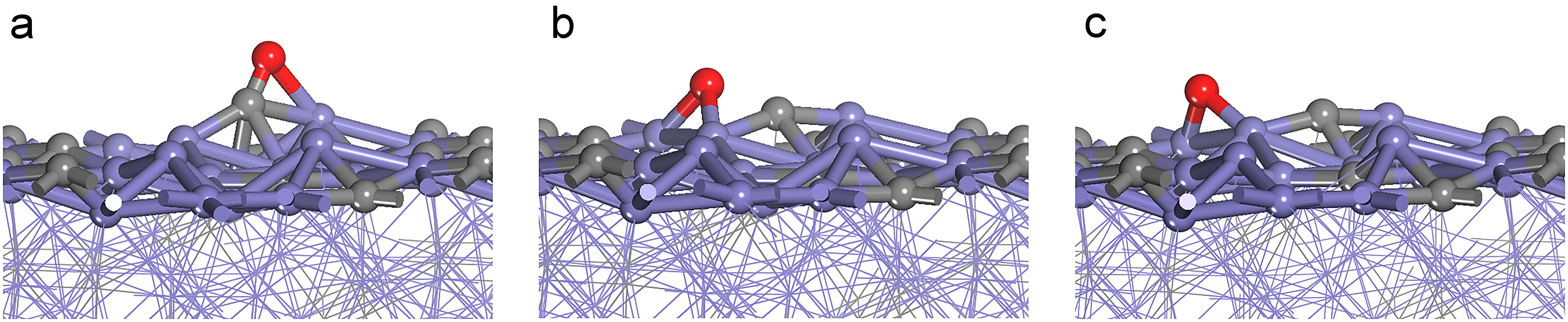
**

**Supplementary Figure 33 |** **Calculated structure models of the direct CO dissociation route on the χ-Fe_5_C_2_(112) facet.** Models of (**a**) CO*, (**b**) TS, and (**c**) C* + O*.


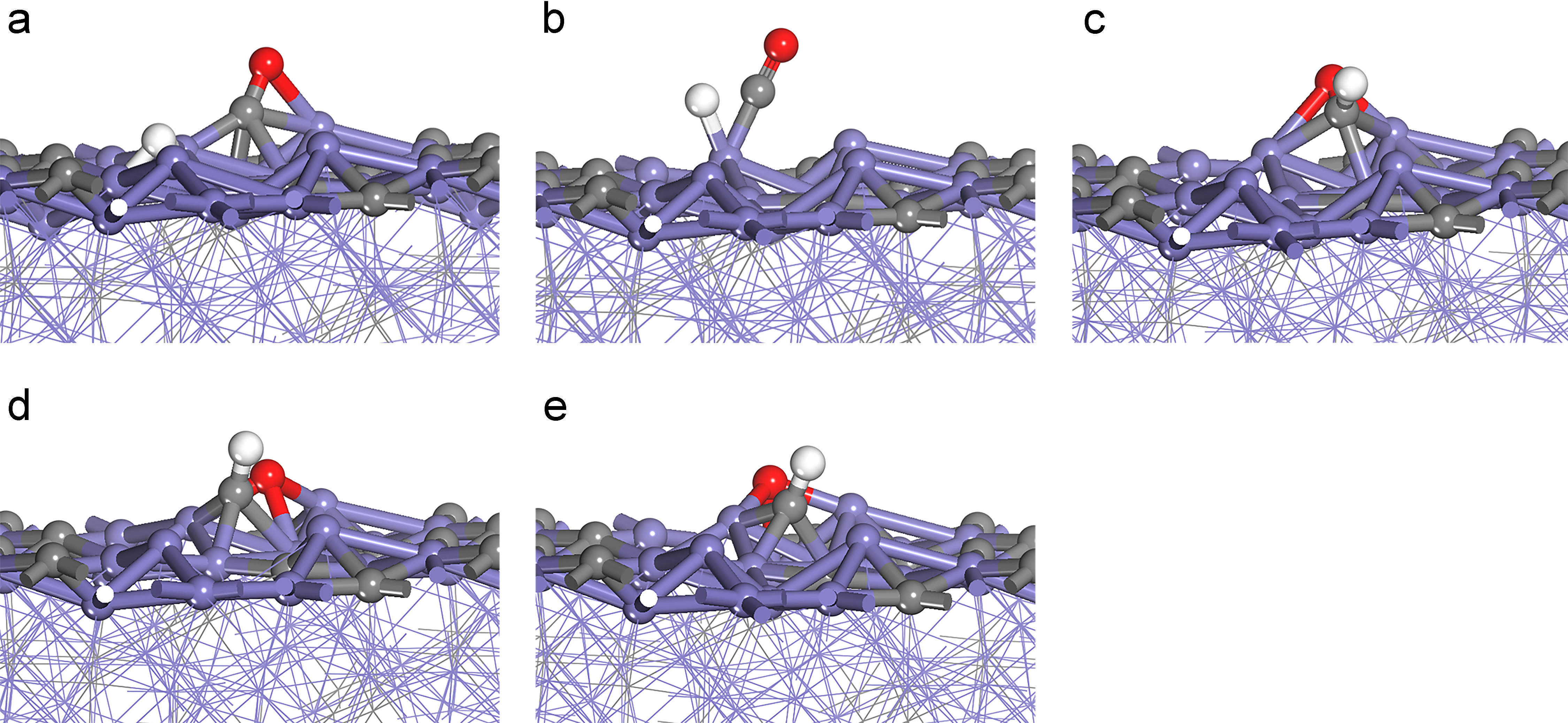


**Supplementary Figure 34 |** **Calculated structure models of the hydrogen-assisted CO dissociation route on the χ-Fe_5_C_2_(112) facet.** Models of (**a**) CO* + H*, (**b**) TS1, (**c**) HCO*, (**d**) TS2, and (**e**) HC* + O*.


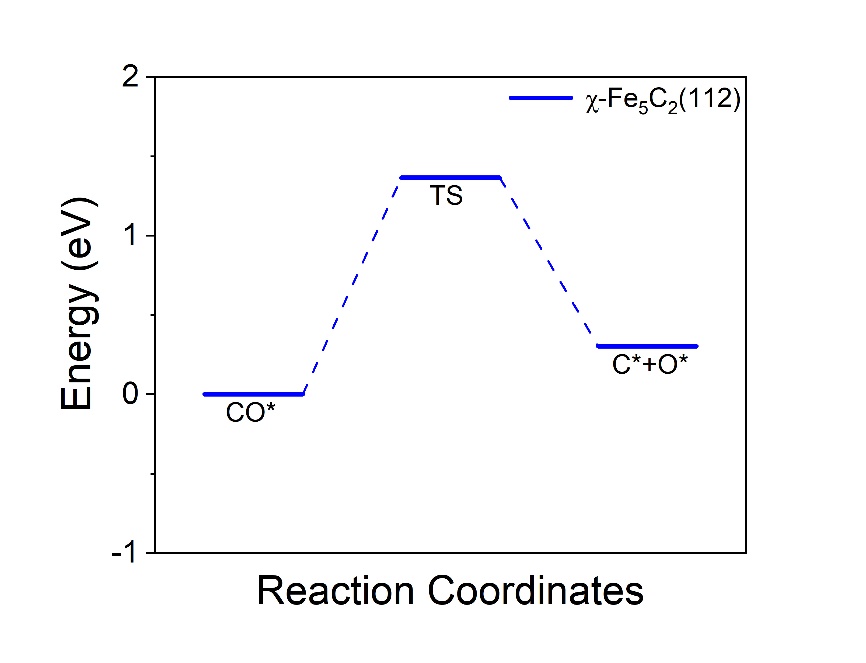


**Supplementary Figure 35 |** **Energy barrier of the direct CO dissociation route on the χ-Fe_5_C_2_(112) facet.**

**
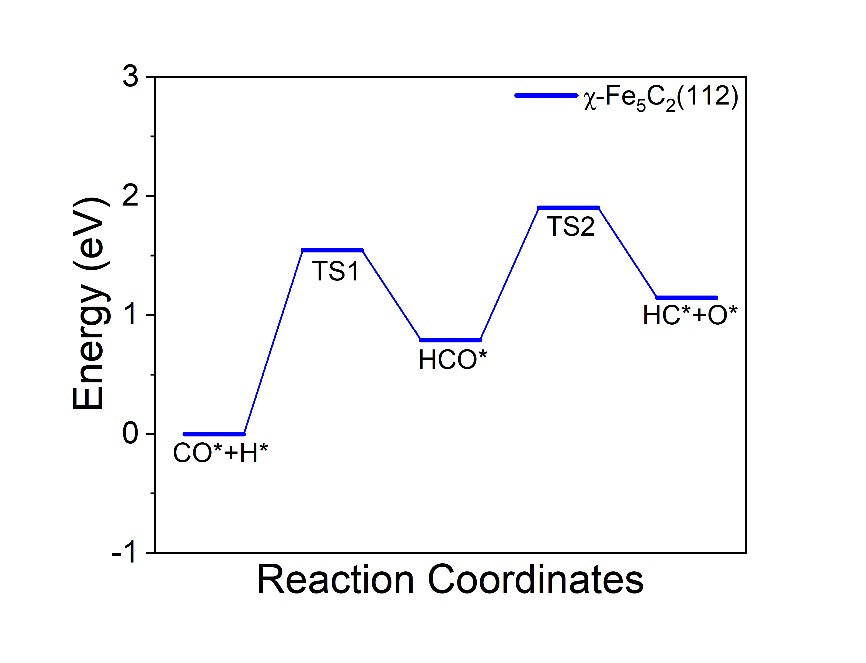
**

**Supplementary Figure 36 |** **Energy barrier of the hydrogen-assisted CO dissociation route on the χ-Fe_5_C_2_(112) facet.**


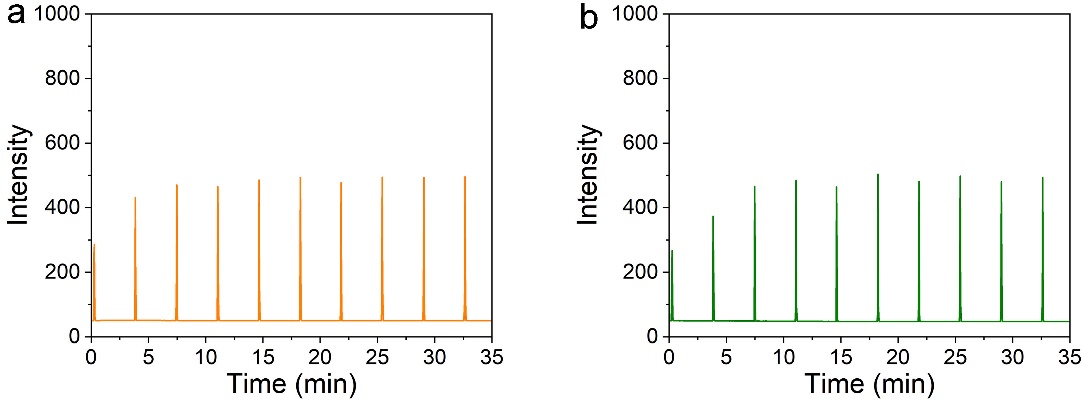


**Supplementary Figure 37 |** H_2_ pulse profiles of (**a**) Fe_3_O_4_@χ-Fe_5_C_2_ nanocubes/SiC and (**b**) Fe_3_O_4_@χ-Fe_5_C_2_ octahedra/SiC.


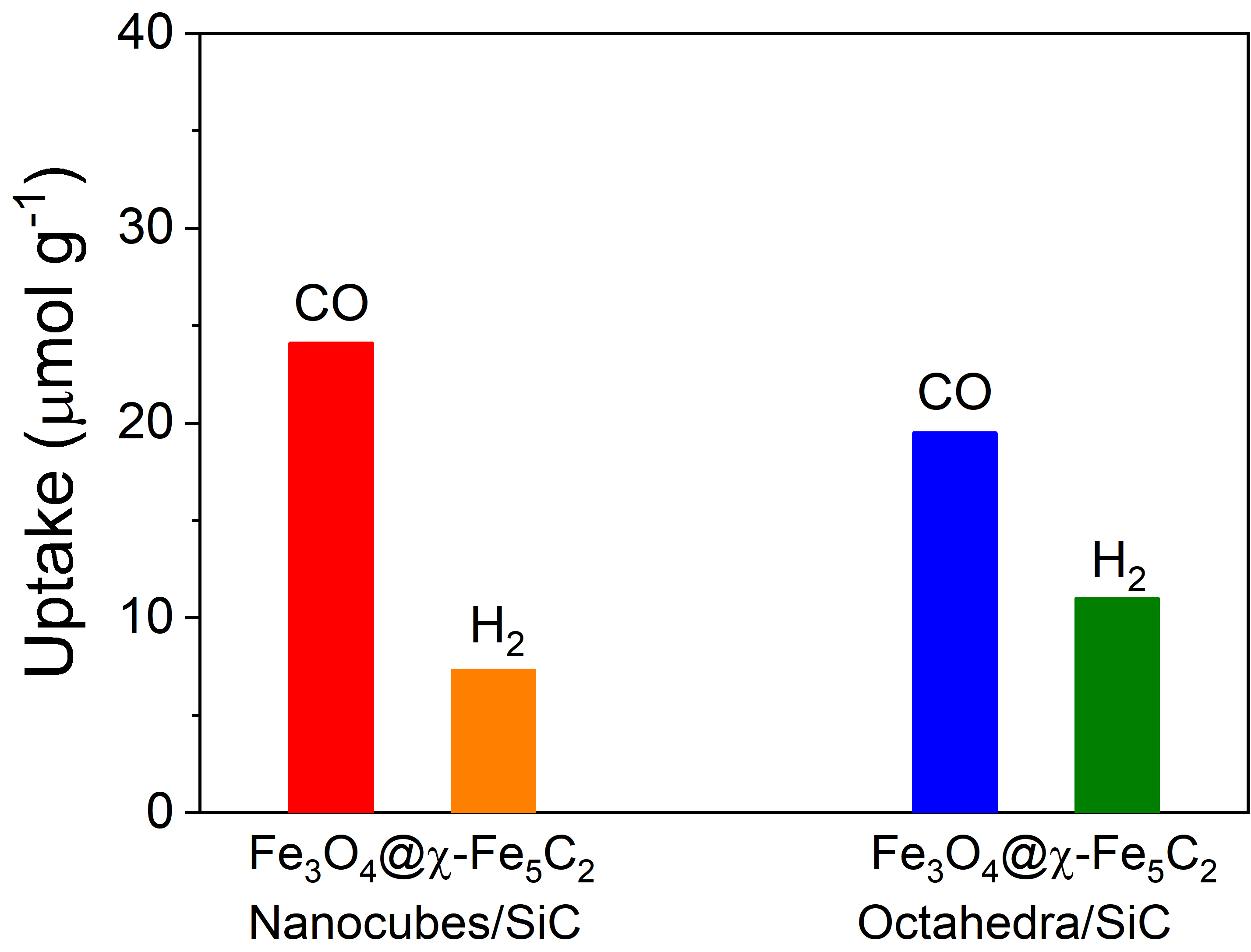


**Supplementary Figure 38 |** The amount of adsorbed CO and H_2_ over Fe_3_O_4_@χ-Fe_5_C_2_ nanocubes/SiC and Fe_3_O_4_@χ-Fe_5_C_2_ octahedra/SiC.

**
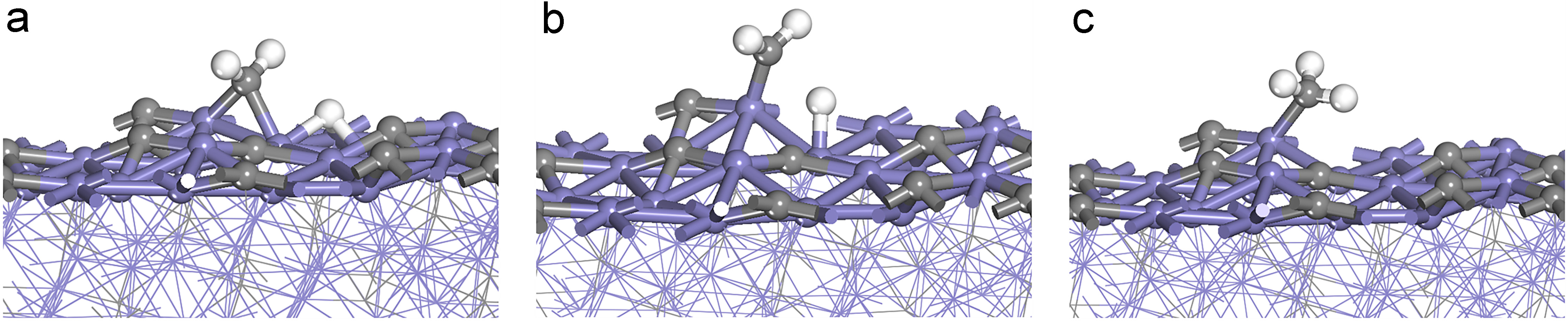
**

**Supplementary Figure 39 | Calculated structure models of CH_2_*+H* on the χ-Fe_5_C_2_(202) facet.** Models of (**a**) CH_2_*+H*, (**b**) TS1, and (**c**) CH_3_*.


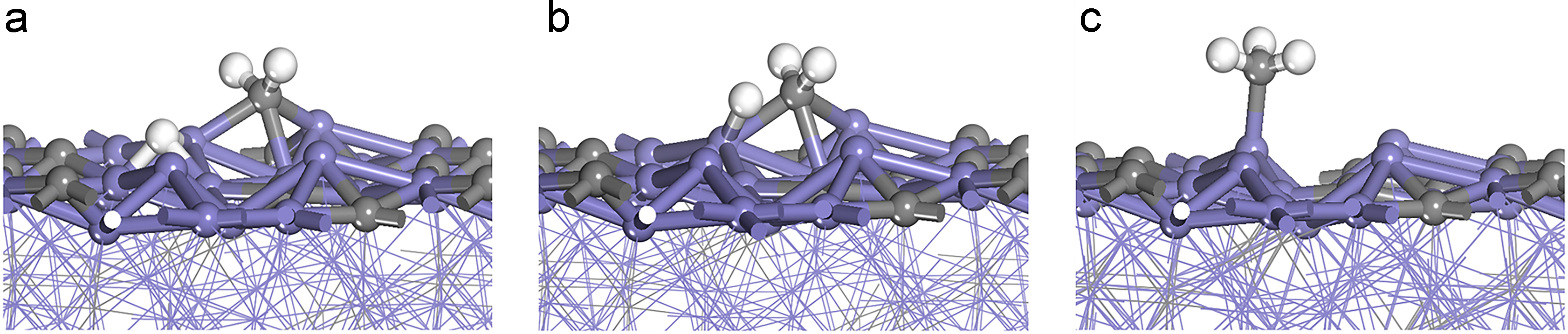


**Supplementary Figure 40 | Calculated structure models of CH_2_* + H* on the χ-Fe_5_C_2_(112).** Models of (**a**) CH_2_* + H*, (**b**) TS2, and (**c**) CH_3_*.

**
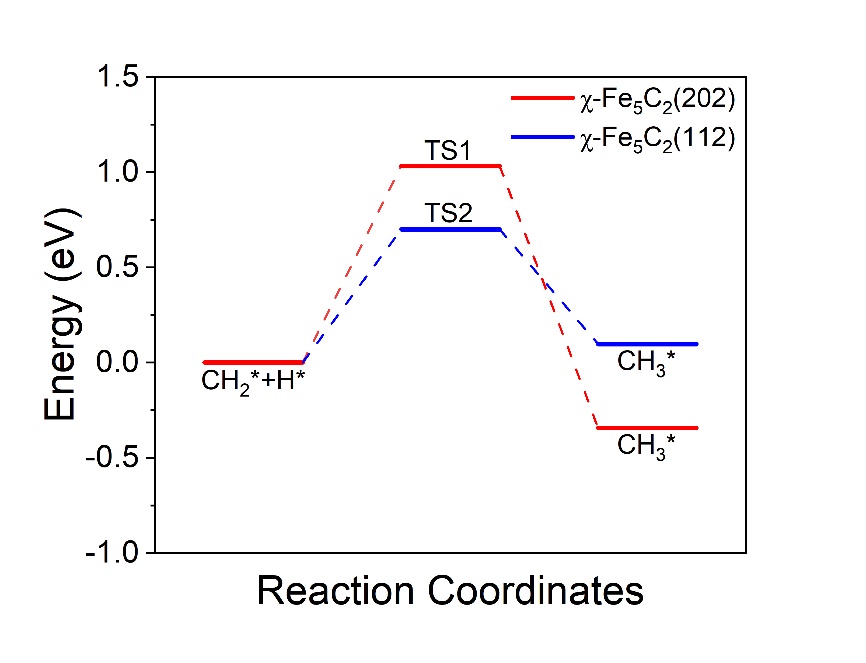
**

**Supplementary Figure 41 | Comparison in energy barriers of CH_2_* + H* over χ-Fe_5_C_2_(202) and χ-Fe_5_C_2_(112) facets.**


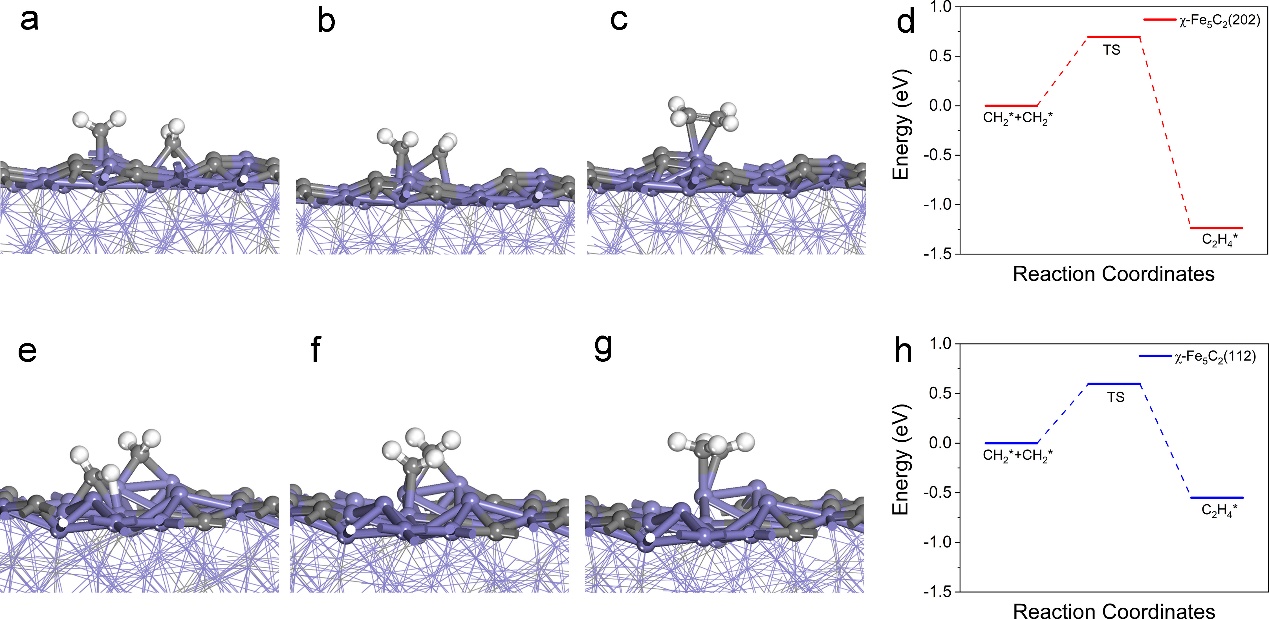


**Supplementary Figure 42 | Calculated structure models and energy barriers of CH_2_*+CH_2_*.** Models of (**a**) CH_2_*+CH_2_*, (**b**) TS, (**c**) C_2_H_4_* over χ-Fe_5_C_2_(202). (**d**) Energy barriers of CH_2_* + CH_2_* over χ-Fe_5_C_2_(202). Models of (**a**) CH_2_*+CH_2_*, (**b**) TS, (**c**) C_2_H_4_* over χ-Fe_5_C_2_(112). (**d**) Energy barriers of CH_2_* + CH_2_* over χ-Fe_5_C_2_(112).

Supplementary References

1. Khan, M. U.; Wang, L.; Liu, Z.; Gao, Z.; Wang, S.; Li, H.; Zhang, W.; Wang, M.; Wang, Z.; Ma, C.; Zeng, J. Pt_3_Co octapods as superior catalysts of CO_2_ hydrogenation. *Angew. Chem. Int. Ed.* **2016**, *55*, 9548-9552.
2. McNab, A. I.; McCue, A. J.; Dionisi, D.; Anderson, J. A. Quantification and qualification by *in-situ* FTIR of species formed on supported-cobalt catalysts during the Fischer-Tropsch reaction. *J. Catal.* **2017**, *353*, 286-294.
3. Huynh, H. L.; Zhu, J.; Zhang, G.; Shen, Y.; Tucho, W. M.; Ding, Y.; Yu, Z. Promoting effect of Fe on supported Ni catalysts in CO_2_ methanation by in situ DRIFTS and DFT study. *J. Catal.* **2020**, *392*, 266-277.
4. Han, X.; Zhao, Q.; Gong, H.; Wei, C.; Lv, J.; Wang, Y.; Wang, M.-y.; Huang, S.; Ma, X. Interface-induced phase evolution and spatial distribution of Fe-Based catalysts for Fischer-Tropsch synthesis. *ACS Catal.* **2023**, *13*, 6525-6535.
5. Bahri, S.; Pathak, S.; Upadhyayula, S. Transient HCO/HCOO^−^ species formation during Fischer-Tropsch over an Fe-Co spinel using low Ribblet ratio syngas: a combined operando IR and kinetic study. *Sustain. Energy Fuels* **2023**, *7*, 708-726.
6. Miao, B.; Ma, S. S. K.; Wang, X.; Su, H.; Chan, S. H. Catalysis mechanisms of CO_2_ and CO methanation. *Catal. Sci. Technol.* **2016**, *6*, 4048-4058.
7. Hartman, T.; Geitenbeek, R. G.; Whiting, G. T.; Weckhuysen, B. M. Operando monitoring of temperature and active species at the single catalyst particle level. *Nat. Catal.* **2019**, *2*, 986-996.
